# A two-stage multi-objective optimization framework for coordinated EV charging scheduling and reactive power dispatch

**DOI:** 10.1038/s41598-026-45109-9

**Published:** 2026-04-15

**Authors:** Mohamed Sayed Badr, H. M. Sharaf, Ahmed M. Zobaa

**Affiliations:** Electrical Power Engineering Department Faculty of Engineering, Cairo University, Giza, 12613 Egypt

**Keywords:** Electric vehicles, EV charging patterns, Multi-objective optimization, Reactive power dispatch, Grid metrics, Ozone layer depletion, Energy science and technology, Engineering

## Abstract

Car exhaust emissions significantly contribute to the depletion of the ozone layer. Electric vehicles (EVs) present a sustainable alternative to mitigate this environmental issue. However, the large-scale adoption of EVs introduces challenges for the power grid, primarily due to irregular and uncoordinated charging patterns. This study proposes a comprehensive two-stage framework for optimizing electric vehicle (EV) charging patterns and reactive power dispatch within power distribution systems. In Stage 1, two types of EV charging schedules are developed and compared: day-ahead charging and real-time charging. Day-ahead charging involves planning EV charging over a 24-hour horizon with the objective of minimizing load variance, energy cost, active power losses, and voltage drop, while simultaneously maximizing voltage stability. Real-time charging dynamically adjusts charging behavior based on immediate grid conditions to minimize load variance and charging costs. Stage 2 focuses on optimal real-time reactive power dispatch, utilizing the reactive power capabilities of EV inverters to further reduce the active and reactive power losses. Additionally, the study analyzes EV behavior in response to sudden load changes, providing critical insights for enhancing grid performance. Different optimization algorithms are implemented to efficiently solve the proposed models, including particle swarm optimization, dandelion optimization, wild horse optimization, and slime mould optimization. The optimization is formulated as a multi-objective problem to consider both grid constraints and customer satisfaction. The proposed framework is applied and tested on a 33-bus radial distribution system with 984 electric vehicles using MATLAB M-files, while power flow calculations are performed using the MATPOWER toolbox. Simulation results demonstrate the effectiveness of the proposed framework. Daily active power losses are reduced from 4.04 MWh to 2.55 MWh and 2.77 MWh under day-ahead and real-time planning strategies—representing reductions of 36.8% and 31.4%, respectively. Similarly, EV charging costs drop from 552.31 USD to 394.19 USD and 363.68 USD, achieving cost savings of 28.63% and 34.15%. Furthermore, voltage profiles are maintained within the acceptable operational limit of 0.95 p.u. These outcomes highlight the significant advantages of the proposed methodology in enhancing grid efficiency while ensuring user satisfaction.

## Introduction

### Motivation

Electric vehicles (EVs) have garnered significant attention in recent years due to their potential to substantially reduce long-term environmental impacts. By eliminating harmful greenhouse gas emissions, EVs contribute to reducing dependency on fossil fuels^1^. However, their widespread use with uncoordinated or random charging introduces several challenges to distribution systems, including increased peak demand, higher energy losses, and voltage violations^2^ as existing grid infrastructure was not originally designed to accommodate the heavy load imposed by high EV penetration. These challenges have motivated researchers to develop effective solutions to mitigate the adverse impacts on grid stability while maximizing the economic benefits for users.

### Literature review

Coordinated charging strategies of EVs typically involve aggregators that optimize EV charging schedules to enhance grid performance and maintain user satisfaction^3–7^. The problem can be formulated as an optimization problem with objectives and constraints. A single objective function that minimizes the total energy demand is proposed in^3^. However, it neglects the charging cost reduction achieved through optimization. In general, single objective models often fall short in balancing the requirements of both grid operators and EV users. To address these limitations, multi objective function is implemented in^4^ to minimize power losses, peak demand, and charging cost using an interrupted fixed-charging strategy, while in^5^ maximizing loadability, the voltage stability index, and minimizing active power losses. Alternatively, some EV charging coordination strategies are non-optimization-based, relying instead on rule-based approaches such as if–then control techniques, as reported in^6^. The EVs’ coordination can be carried out through either a day-ahead or a real-time charging schedule. In the day-ahead approach, optimal charging schedules for all EVs are generated one day in advance, based on forecasted EVs data, as predicted arrival and departure times for all vehicles, and are then applied as the charging plan for the following day^3–7^. In contrast, the real-time approach determines the optimal charging schedule dynamically within each time slot of the day, for only newly arrived EVs, as discussed in^8^.

**Fig. 1:**
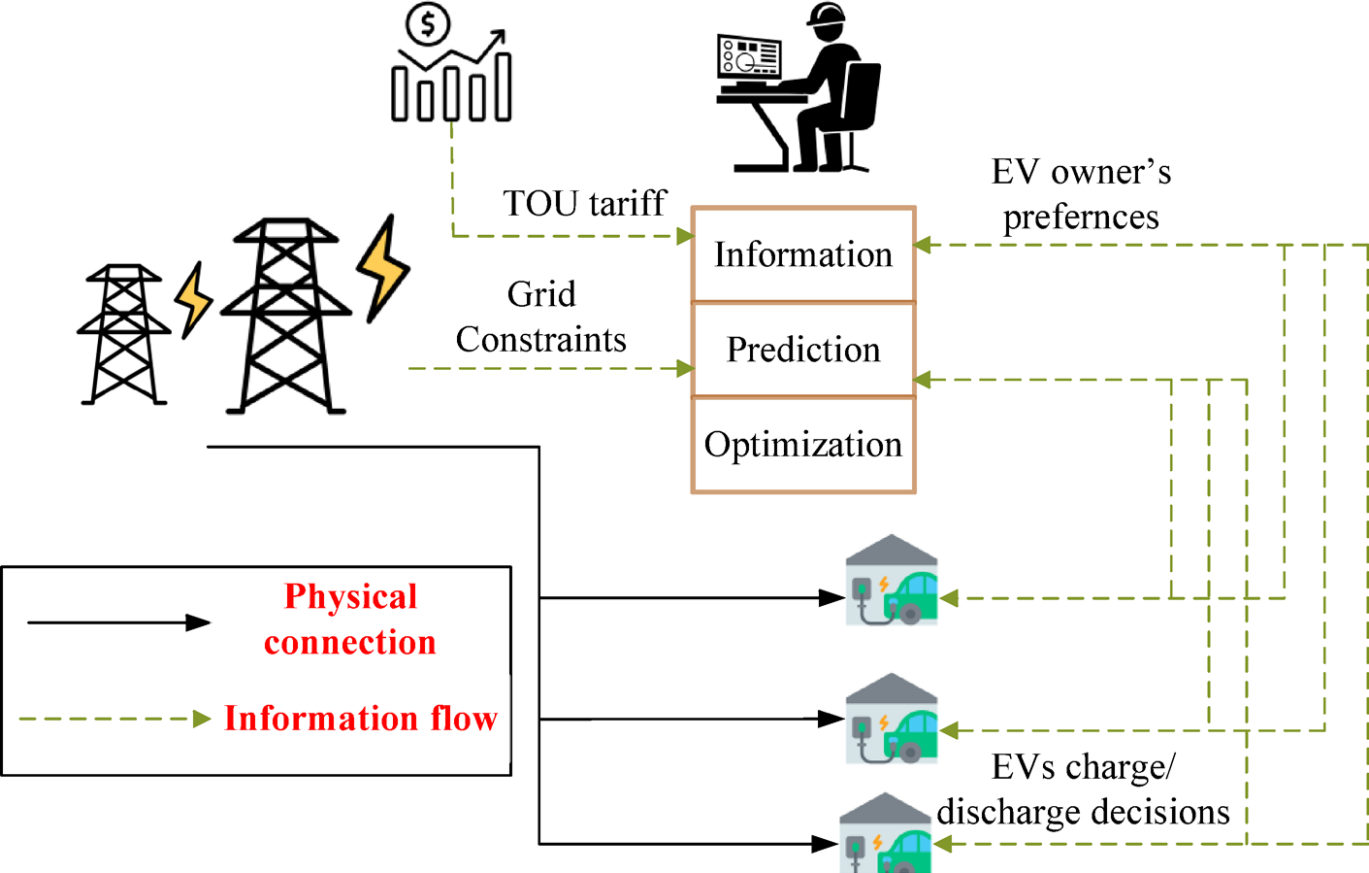
Centralized aggregator.

EV charging control architectures may be implemented in either a centralized or a decentralized manner. In the centralized approach, as shown in Fig. 1, An aggregator collects data from all EVs and determines an optimal charging schedule. Many studies^3–6^ have adopted this centralized approach. While effective, it becomes computationally demanding as the number of EVs grows. In the decentralized approach, each user’s controller independently decides when to charge; however, this method does not guarantee an optimal solution for the overall system. A decentralized control strategy is employed in^7^, where each EV’s charging power is adaptively regulated in real-time using droop-based techniques, based on local measurements of nodal voltage levels, voltage imbalance, and the EV’s state of charge (SOC). While coordinated charging mitigates several operational issues, it primarily focuses on active power scheduling and does not fully exploit the bidirectional capability of EVs inverter.

**Fig. 2:**
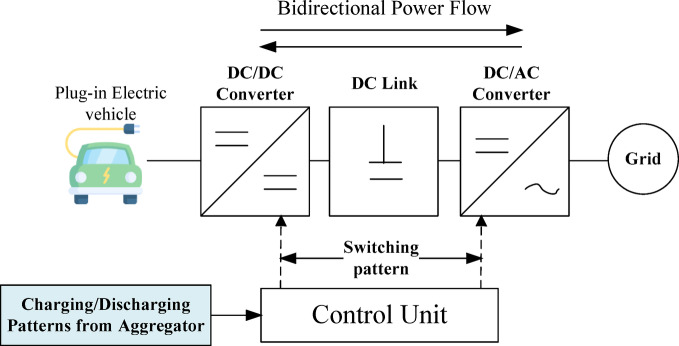
V2G technology.

EVs can exchange power with the grid in two modes: Grid-to-Vehicle (G2V) and Vehicle-to-Grid (V2G), as illustrated in Fig. 2. In the V2G mode, EVs function as distributed energy storage systems, discharging power during peak demand and charging during low-load periods, thereby supporting peak shaving and valley filling. A recent review identified 131 V2G projects implemented across 27 countries by December 2023, with notable activity in Europe (UK, Netherlands, and Denmark), the USA, and Asia (particularly China), as well as emerging initiatives in Australia^9^. Extensive research has modelled V2G and G2V within an optimization framework, in which the charging and discharging profiles of EVs are scheduled to achieve predefined operational objectives while satisfying both system and user constraints. An optimization framework for the optimal charging and discharging of EVs, integrated with a hydrogen storage system (HSS), is presented in^10^ to maximize profit. An optimization model is developed in^11^ to reduce the cost difference between charging costs and discharging revenues. A decentralized scheduling technique is introduced in^12^, where each EV determines it’s charging and discharging behavior locally while coordinating with an aggregator to flatten load curve. A critical oversight in many studies^10–12^ is the exclusion of battery degradation costs due to discharging power to the grid. Frequent charging and discharging cycles accelerate battery aging and reduce its lifespan^13^. To address this, a coordinated charging/discharging algorithm is designed in^8^, which minimizes load variance and overall operational cost, while explicitly accounting for battery degradation cost in the objective function, in addition to charging cost and discharging revenue. Another important role of V2G is its capability to reduce Energy Not Supplied (ENS) and operating costs, in dynamic network reconfiguration (DNR) as reported in^14–16^. In^17^ a real-time EV routing and energy-aware transportation framework for smart cities is presented, integrating traffic conditions, charging station availability, and V2G incentives. However, this study mainly focuses on transportation networks, with limited emphasis on coordinated charging schedule.

Reactive power management is essential for voltage regulation and network performance enhancement^18^. In addition to active power management, V2G systems can inject or absorb reactive power, which plays a crucial role in voltage regulation. Furthermore, the injection or absorption of reactive power does not impact the battery’s state of charge (SOC) or its lifespan^19^. However, this functionality has not been thoroughly investigated in previous studies, leading to several voltage-related limitations. Specifically, some works fail to maintain the voltage within the acceptable limits ± 0.95 p.u^4,10^, others do not mention the voltage profile at all^3,8,11^, and some rely on alternative reactive power sources, such as capacitor banks, for voltage correction^4,20^.

By utilizing the reactive power capability of EVs, voltage levels can be maintained within the limits ± 0.95 p.u. A V2G-based control strategy is designed in^21^ to optimize both active and reactive power flow, with the objectives of decreasing voltage-dependent load consumption and reducing network losses. Another V2G control strategy is presented in^22^, which not only regulates active and reactive power but also utilizes the inverter’s capability to inject harmonic currents, thereby reducing both power losses and total harmonic distortion (THD). While^21,22^ provide significant advancements in grid support via EVs, they focused on optimizing power flows at the node level without addressing coordination at the individual EV level. Additionally, these studies do not consider the impact of battery degradation. A two-stage strategy is formulated in^23^. In the first stage, individual EV charging patterns are optimized without discharging active power, so as to avoid battery degradation, with the goal of minimizing load variance. The results from this stage serve as input to the second stage, which optimizes reactive power dispatch at each node and adjusts tie-switch positions through distribution network reconfiguration (DNR) to minimize active power losses. A single-objective optimization function is developed in^24^ to optimally dispatch reactive power from medium- and heavy-duty (MDHD) electric vehicles, with the aim of reducing voltage deviations at each bus in the distribution network^25^. is proposed a single-objective function to optimize the reactive power dispatch of EV charging stations, aiming to maximize voltage stability in the distribution network. A two-stage approach for EV planning is presented in^26^. The first stage focuses on optimizing EV charging schedules to reduce load variance, while the second stage targets the optimization of EV reactive power dispatch. A subsequent cost-benefit analysis is then conducted. In^27^ the framework begins with the aggregator optimizing EV charging and discharging to maximize profit, ignoring active power curtailment. Then, the DSO performs a power flow analysis to determine the reactive power needed for voltage improvement and calculates the resulting active power curtailment. This is sent back to the aggregator, which reoptimizes considering the curtailment effect.

Renewable energy sources, such as photovoltaic (PV) and wind systems, are often suggested for integration with energy storage systems, including hydrogen storage and electric vehicle (EV) due to their complementary operational characteristics^28,29^. EVs can absorb excess renewable generation during periods of high supply and feed power back to the grid during demand peaks, thereby improving renewable energy utilization, reducing curtailment, and enhancing grid stability. A methodology based on fuzzy control is presented in^30^, focusing on modelling the stochastic behaviour and continuous variations of wind power. To manage excess energy and mitigate uncertainties in wind generation, electric vehicles and superconducting magnetic energy storage (SMES) systems are used. In^31^ EVs are modeled as flexible loads to mitigate renewable energy variability and enhance supply–demand balance within the system.

Renewable energy resources, such as photovoltaic (PV) and wind power systems, operate in a manner similar to electric vehicles (EVs) by utilizing bidirectional inverters. These inverters are capable of both injecting and absorbing reactive power for voltage regulation. A Three-stage Model Predictive Control (MPC) framework is presented in^32^ comprising transformer tap control (hourly), reactive power dispatch by EVs and PV (every minute), and active power scheduling (every 30 min) based on real-time pricing (RTP). A multi-objective optimization approach is proposed in^33^, targeting line loss minimization, voltage deviation minimization, and static voltage stability margin maximization with decision variables the reactive power dispatch for both EVs and renewable energy sources (PV and wind). A multi-objective optimization is proposed in^34^ to minimize both system power losses and voltage drop with decision variables, tie switches placement (DNR), and the reactive power dispatch for both EVs and renewable energy sources (PV and wind). However, both^33,34^ lacked simulations over a full day-ahead load variation cycle, limiting the validation of the proposed methods under realistic operating conditions and grid changes.

### Research gaps

According to Table 1, several research gaps can be identified. Although extensive research has been conducted on electric vehicle charging coordination, critical limitations remain in the existing literature. Most previous studies primarily focus on day-ahead scheduling frameworks, with limited consideration of real-time operational variations, and fail to adequately address real-time reactive power management. Moreover, the uncertainty in EV behavior is often neglected, which reduces the robustness of existing approaches under practical operating conditions. In addition, many existing approaches lack Pareto-based multi-objective analysis to adequately capture the trade-offs among conflicting objectives. Furthermore, the computational complexity and scalability of proposed algorithms are rarely analyzed, which raises concerns regarding their practical implementation in large-scale distribution networks.

**Table 1:** Summary of research gaps in existing EV charging coordination studies.

Ref.	Day-ahead charging	Real-time charging	V2G active power discharging	EV inverter reactive power support	Voltage constrains	Cost reduction	Complexity analysis	Pareto set
^3^	✓	✗	✗	✗	Not mentioned	✗	✗	✗
^4^	✓	✗	✗	✗	± 10%	✓	✗	✗
^5^	✓	✗	✗	✗	± 10%	✗	✗	✗
^8^	✗	✓	✓	✗	Not mentioned	✗	✗	✗
^10^	✓	✗	✓	✗	± 10%	✓	✗	✗
^11^	✓	✗	✓	✗	Not mentioned	✓	✗	✗
^21^	✓	✗	✓	✓	± 5%	✗	✗	✗
^22^	✓	✗	✓	✓	± 5%	✓	✗	✗
^23^	✗	✓	✗	✓	± 5%	✗	✗	✗
^26^	✓	✗	✓	✓	± 10%	✓	✗	✗
Current paper	✓	✓	✗	✓	± 5%	✓	✓	✓

### Paper contribution

**Fig. 3:**
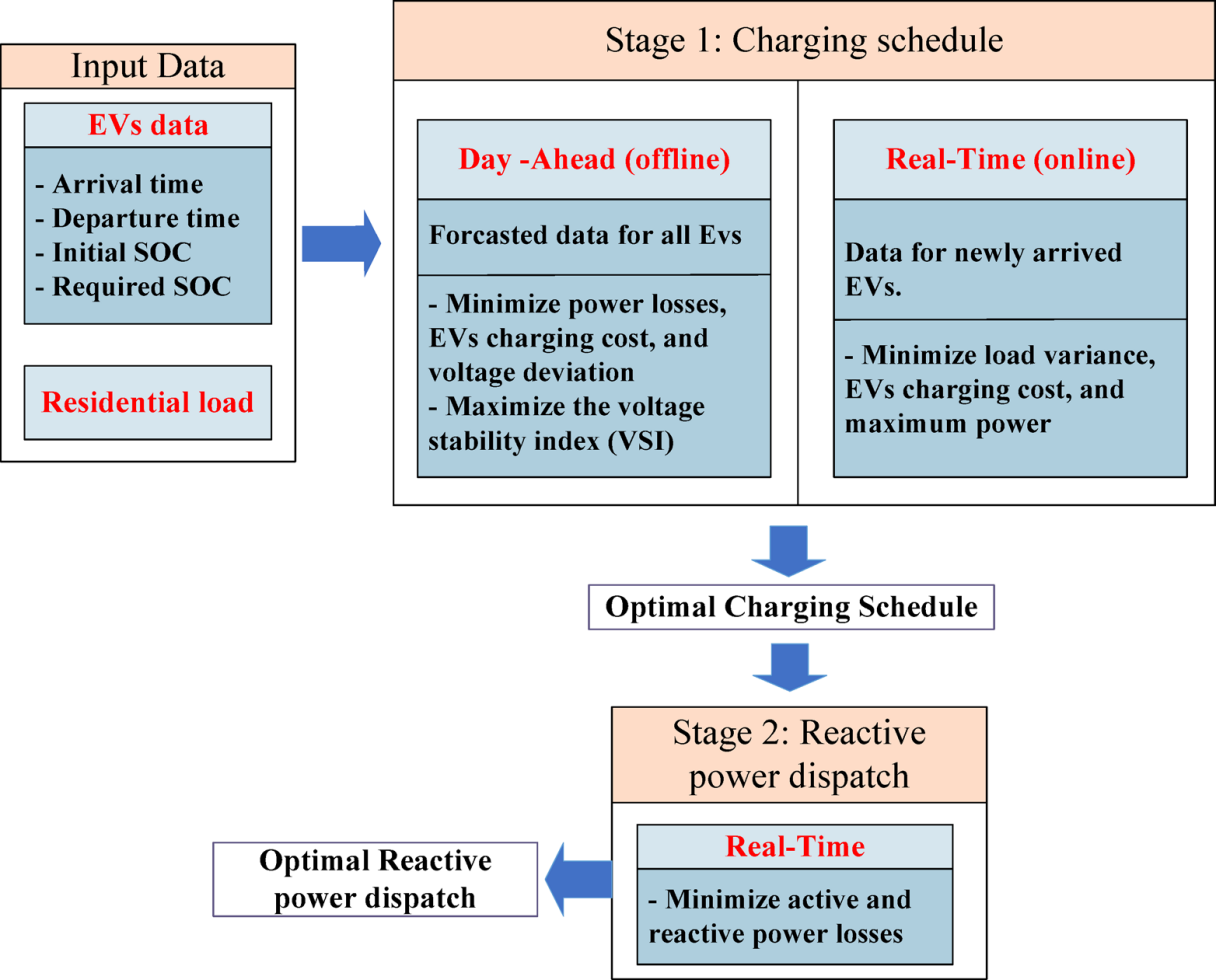
Architecture of the proposed two-stage optimization framework.

This study addresses the operational challenges arising from unpredictable, uncoordinated EV charging and their impact on distribution network performance. To overcome these challenges, a structured two-stage optimization framework is proposed, as illustrated in Fig. 3. In the first stage, optimal charging decisions are determined by developing and comparing two charging strategies: a day-ahead plan and a real-time plan. In the second stage, a real-time reactive power dispatch model is formulated to ensure voltage stability and minimize active and reactive power losses under network constraints. The key contributions of this research can be outlined as follows:

- This study investigates the challenges posed by unpredictable and uncoordinated EV charging on distribution network performance.
- A two-stage coordinated framework for EV charging scheduling and reactive power management is developed:
  - Stage 1: Optimal Charging Schedules: Designed two types of EV charging strategies:
    Day-ahead charging schedules and Real-Time charging schedules.
  - Stage 2: Optimal Real-Time Reactive Power Dispatch.
- Multiple optimization algorithms are implemented to efficiently solve the proposed problems, ensuring robustness, scalability, and computational effectiveness under varying system conditions.
- The effectiveness of the proposed framework is validated through a comprehensive case study conducted on the IEEE 33-bus radial distribution system over a full-day time horizon.
- The response of EVs is analysed when the grid is subjected to overvoltage caused by sudden load cuts.
- Uncertainties in EV behaviour are explicitly modelled and systematically tested within the real-time charging framework.

The rest of this paper is structured as follows: Section 2 introduces the operating Mechanism of EV charger. Section 3 presents the proposed methodology. Section 4 describes the case study setup. Section 5 discusses the simulation results. Finally, Section 6 concludes the paper and outlines directions for future work.

## Operating mechanism of residential EV chargers

In this study, EVs are assumed to connect to the distribution network through slow chargers, which are typical of residential (home) charging. When an EV arrives, it provides its expected departure time ($\:{T}_{{Deprature}_{\:i}}$) along with the required state of charge ($\:{SOC}_{{req}_{i}}$) at departure. The charger’s control unit records the arrival time ($\:{T}_{{Arrival}_{\:i}}$), the initial state of charge ($\:{SOC}_{{in}_{i}}$), and key battery parameters, such as charging power ($\:{P}_{{Nominal}_{i}}$) and battery capacity ($\:{E}_{\mathrm{i}})$.

The aggregator begins by calculating the energy requirement for each EV, which is the first step in estimating the overall energy demand of all connected EVs. To compute the charging energy of each EV ($\:{CE}_{\mathrm{i}}$) as mentioned in Eq. (1). Once the charging energy requirement is determined, the charging duration of each EV ($\:{CD}_{\mathrm{i}}$) is computed using Eq. (2). To facilitate precise scheduling, the charging duration is further converted into discrete time slots. Eqn. (3) defines this conversion, in which the 24-hour day is divided into 288 time slots by segmenting each hour into 12 5-minute intervals, enabling fine-grained scheduling of optimal energy distribution.

1 $$\:{CE}_{\mathrm{i}}=\frac{{SOC}_{{req}_{i}}-{SOC}_{{in}_{i}}}{{\upeta\:}}x{E}_{\mathrm{i}}\left(kwh\right)$$

2 $$\:{CD}_{\mathrm{i}}=\frac{{CE}_{\mathrm{i}}}{{P}_{{Nominal}_{i}}}$$

3 $$\:T=\frac{{CD}_{\mathrm{i}}}{5}x60$$

**Fig. 4:**
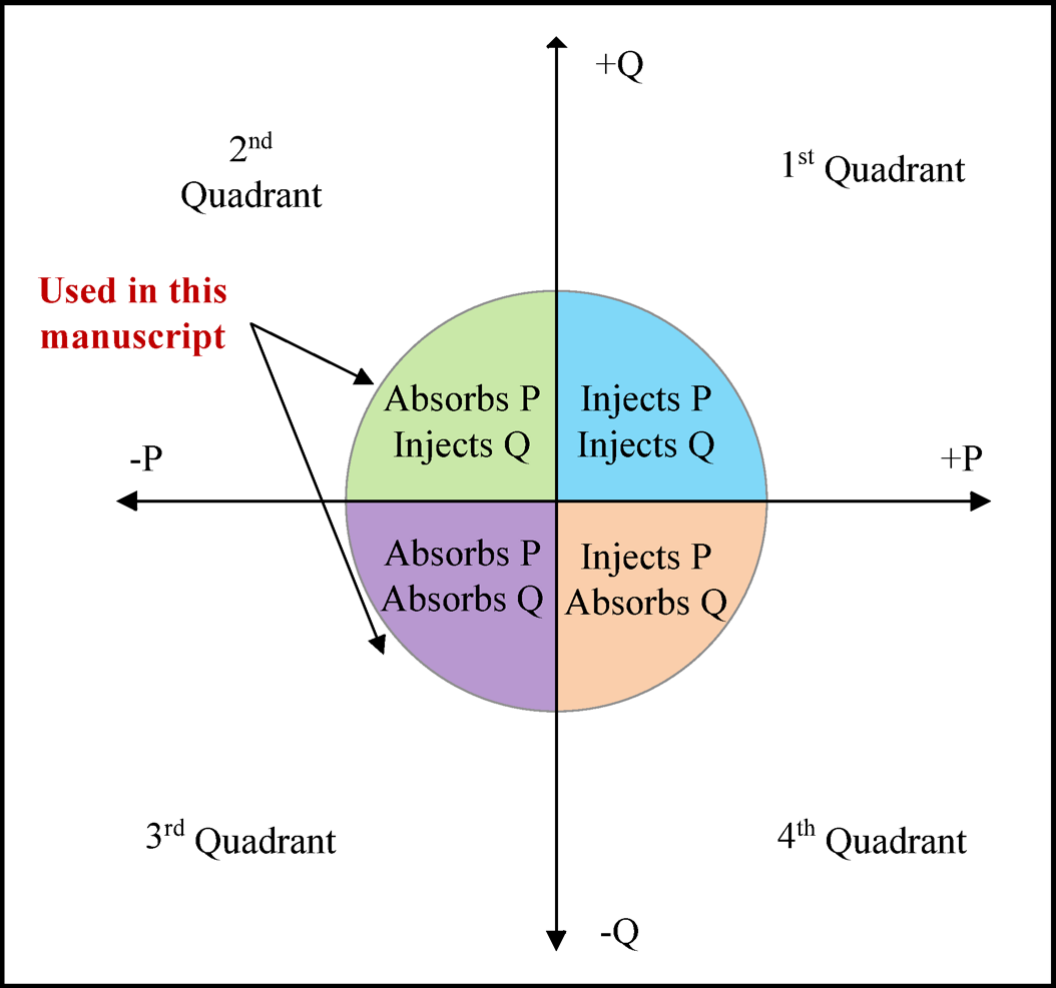
Bidirectional charger operating modes.

The EVs are equipped with bidirectional chargers capable of both injecting and absorbing active and reactive power. When the grid requires reactive power compensation, the EV charger can operate in capacitive mode to supply reactive power or in inductive mode to absorb it, as illustrated in Fig. 4. In this study, EVs are considered to operate in charging mode only, to extend battery lifespan^23^. Therefore, the inverters in Fig. 4 operate in quadrants two and three only.

## Proposed methodology for the optimal charging schedule and reactive power dispatch

A comprehensive mathematical model of the proposed two-stage optimization problem is developed in this section. **A centralized aggregator** is responsible for executing both stages. The first stage addresses the optimal charging schedule of EVs. The second stage focuses on the optimal reactive power dispatch of EVs.

### First stage optimization

The primary goal of this optimization stage is to establish an effective charging schedule for EVs. The optimization can be carried out through either **a day-ahead or a real-time** charging schedule. In the day-ahead approach, optimal charging schedules are generated one day in advance, based on forecasted EVs data, as predicted arrival and departure times for all vehicles, and are then applied as the charging plan for the following day. With full-day data available, this optimization problem can be solved as a network-constrained optimization in which the AC load flow is embedded to evaluate the objective and enforce grid-level constraints (e.g., feeder losses, bus voltages, voltage-stability indices, and line/transformer loading) as used in^4,21^ and^22^.

In contrast, the real-time approach determines the optimal charging schedule dynamically within each time slot of the day, for only newly arrived EVs, as discussed in^8^ and^23^. Owing to the absence of full-horizon EV information, embedding load-flow analysis becomes impractical and computationally prohibitive. Consequently, the objective functions are evaluated using mathematical formulations. While real-time approaches offer greater flexibility and can handle unplanned EV arrivals, their myopic decision-making nature prevents them from guaranteeing a globally optimal solution for the entire grid. A detailed comparison between day-ahead and real-time charging strategies is presented in Table 2.

**Table 2:** Comparison between day-ahead and real-time charging schedules.

Aspect	Day-ahead (DA)	Real-time (RT)
Optimization applied	Applied once on the previous day to plan for the next day for all EVs	Applied at each time slot for newly arrived EVs only, so it runs up to 288 times/day
Requirements	Require next day forecasted data of EVs and grid	Require only the EVs and grid data of current time slot
No of control variables	Number of all EVs	Number of currently arrived EVs only
Advantages	-Can provide a globally optimal solution for the full scheduling horizon Network-constrained optimization with embedded AC load flow Reduces computational requirements during the operation day	-Can handle uncertainty and unplanned EV arrivals -More adaptable to real-time conditions
Limitations	-High computational time for large systems -Relies heavily on forecast accuracy; errors in predicted EV arrivals/departures or loads can reduce performance	-Provides a locally optimal solution for the current time slot; may not be globally optimal -AC load flow is not embedded within the optimization loop (validated separately) -Requires continuous execution throughout the day
Selection criteria	-Forecast accuracy of EVs and loads is high -System conditions are relatively stable	-EV behavior is highly uncertain -Distribution networks are sensitive to load fluctuations

In the day-ahead charging schedule, the ac load flow is run at every time slot, for each particle and each iteration. Although this significantly increases the computational burden, the approach remains feasible as it is performed offline. In the real-time charging schedule, the ac load flow is not included inside the optimization loop. Instead, the objective functions are calculated using mathematical formulas. The load flow is executed only once after the optimization is finished at the current time slot to evaluate the network results.

#### Day-ahead charging schedule approach

The day-ahead EV charging strategy schedules charging over a 24-hour horizon using deterministic forecasts such as electricity prices, base load, and EV availability.

- Optimization control variables.
  At this stage of the optimization process, the charging start time of all EVs is considered as the control variable and is used to construct the charging matrix presented in Table 3. This matrix defines the charging status of all EVs over the daily scheduling horizon, which is divided into 288 time slots.
  Each EV is assumed to follow an individual charging profile with constant and continuous charging power throughout its charging duration. Once the charging process is initiated, the EV continues charging without interruption until its required energy demand is fully satisfied. Therefore, the optimization does not control the charging power magnitude, but rather determines the most appropriate charging initiation time for each EV, while respecting system operational constraints and EV-specific requirements. Where $\:{P}_{{nominal}_{1}}$ is the nominal power of EV charger number 1. The charging matrix is then incorporated into the distribution network model to evaluate the objective functions, enforce the operational constraints, and analyze the impact of EV integration on the distribution network.
  **Table 3:**
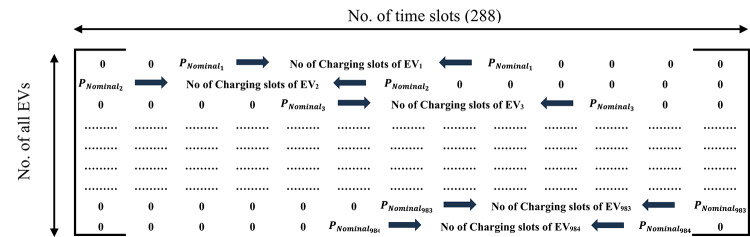
Charging matrix of all EVs throughout the day.
- The multi-objective function consists of:

1. Minimizing the maximum power losses of the system.
  4 $$\:{f}_{1-DA}^{1st}=\sum\:_{i=1}^{{N}_{L}}{\sum\:}_{t=0}^{T}{P}_{{Losses}_{peak}}$$
  Here, $\:{N}_{L}$ represents the number of lines, while T denotes the total number of time slots within 24 h which is equal to 288.
2. Minimizing the total charging cost
  5 $$\:{f}_{2-DA}^{1st}=\sum\:_{i=1}^{{N}_{EV}}{\sum\:}_{t=0}^{T}\mathrm{C}\mathrm{h}\mathrm{a}\mathrm{r}\mathrm{g}\mathrm{i}\mathrm{n}\mathrm{g}\:\mathrm{C}\mathrm{o}\mathrm{s}\mathrm{t}$$
  Here, $\:{N}_{EV}$ denotes the total number of EVs scheduled to arrive on the following day.
3. Minimizing the maximum voltage drop
  6 $$\:{f}_{3-DA}^{1st}=\sum\:_{i=1}^{{N}_{b}}{\sum\:}_{t=0}^{T}{\mathrm{m}\mathrm{a}\mathrm{x}\left(\right({V}_{rated}-{V}_{b})}^{2})$$
  Here, $\:{V}_{rated}\:$ denotes the rated voltage value (1 p.u.), $\:{V}_{b}$ represents the voltage at each node, and $\:{N}_{b}$ is the total number of nodes in the system.
4. Maximizing the voltage stability index (VSI)
  7 $$\:{f}_{4-DA}^{1st}=\sum\:_{i=1}^{{N}_{b}}{\sum\:}_{t=0}^{T}{\updelta\:}\mathrm{m}\mathrm{i}\mathrm{n}$$
  Where $\:{{\updelta\:}}_{min}$ denotes the minimum singular value of the power-flow Jacobian matrix, which is widely used as an indicator of voltage stability. A higher value of $\:{{\updelta\:}}_{min}$ implies improved system stability and better numerical conditioning, whereas a value approaching zero indicates an ill-conditioned or nearly singular matrix.
5. Minimizing the load variance
  8 $$\:{f}_{5-DA}^{1st}=\sum\:_{t=0}^{T}{({P}_{t}^{Load}+\sum\:_{m=1}^{{N}_{EV}}{P}_{t,m}^{EV}-{P}_{Avg}^{T})}^{2}$$
  9 $$\:{P}_{Avg}^{T}=\frac{1}{T}\mathrm{*}{\sum\:}_{t=0}^{T}({P}_{t}^{Load}+{\sum\:}_{m=1}^{{N}_{EV}}{P}_{t,m}^{EV})$$
  Where $\:{P}_{t}^{Load}$ is the base load of the grid at time t, $\:{P}_{Avg}^{T}$ is the average load of the grid in a day, $\:{P}_{t,m}^{EV}$ is the charging power of EV number m at time t.

*Normalization*: Since the five objective functions are expressed in different physical units and exhibit significantly different numerical magnitudes, a normalization step is required before optimization to enable a fair comparison among them.

Several normalization techniques can be employed for this purpose, including min–max normalization, median-based normalization, Z-score normalization, as well as L1-norm and L2-norm normalization^35^. Among these techniques, the min–max normalization is selected as the most suitable approach, as discussed in^35^. This method scales each objective value into the normalized range of [0,1]. Accordingly, min–max normalization is applied to all objective functions, as defined in Eq. (10). $\:{f}_{Calc}$ denotes the value obtained from the optimization, while $\:{f}_{min}$ and $\:{f}_{max}$ represent the lower and upper bounds of the corresponding objective function, respectively. $\:{f}_{normalized}^{{\prime\:}}$ is the normalized value.

10 $$\:{f}_{normalized}^{{\prime\:}}\:=\:\:\frac{{f}_{Calc}-{f}_{min}}{{f}_{{max}}-{f}_{min}}$$

*Scaling*: After normalization, all objective function values lie within the range [0,1]. However, this does not necessarily imply that all objectives have the same order of magnitude. Therefore, an additional scaling step is required to ensure a fair comparison among the objectives^36^. For example, if the normalized objective values are $\:{f}_{1}^{{\prime\:}}$=0.001,$\:{f}_{2}^{{\prime\:}}$=0.3 and $\:{f}_{3}^{{\prime\:}}$=0.7, so $\:{f}_{1}^{{\prime\:}}$=0.001 will be multiplied by 100 to make the value 0.1 to be suitable for comparison with others objectives. Where $\:{f}_{1}^{{\prime\:}}$ is the value after normalization, and $\:{f}_{1}^{{\prime\:}{\prime\:}}$ is the value after normalization and scaling.

The problem is formulated **as a multi-objective optimization problem**. The overall objective function is expressed as a weighted sum of individual objective functions, as given by

11 $$\:Multi\:Objective\:Fun:\:.\left(F\right)=W1*{f}_{1-DA}^{{\prime\:}{\prime\:}1st}+W2*{f}_{2-DA}^{{\prime\:}{\prime\:}1st}+W3*{f}_{3-DA}^{{\prime\:}{\prime\:}1st}+W4*1/{f}_{4-DA}^{{\prime\:}{\prime\:}1st} +W5*{f}_{5-DA}^{{\prime\:}{\prime\:}1st}$$

Weights W1​–W5 indicate the operator’s preference across objectives, each set to 0.2 in this manuscript.

- **Constrains**.

1. Voltage
  Voltage drop is imposed as a constraint across all feeders to ensure it remains within the permissible limit of 10% as in Eq. (12).
  12 $$\:\left|{V}_{rated}-{V}_{b}\right|\le\:0.1$$
2. Grid demand
  This constraint is important to ensure that the demand Load doesn’t exceed the Grid capacity.
  13 $$\:{S}_{transformer}\le\:Maximum \:Grid \:demand$$
3. State of charge limit
  The depth of charge plays a crucial role in determining battery lifetime and may lead to burning^37^. To protect the battery from excessive charging, its state of charge must be carefully controlled within predefined limits, where $\:{SOC}_{{ev}_{i,t}}$ is the current state of charge of EV number i at time slot t^8^.
  14 $$\:{SOC}_{{in}_{i}}\le\:{SOC}_{{ev}_{i,t}}\le\:{SOC}_{{rec}_{i}}$$
4. Starting time of charging
  To guarantee that every EV is fully charged by its departure time, the charging start time of each EV ($\:T{st}_{i}$​) is constrained within a feasible window defined by a lower bound ($\:\mathrm{L}{\mathrm{B}}_{\mathrm{i}}$​) and an upper bound ($\:\mathrm{U}{\mathrm{B}}_{\mathrm{i}}\:$​). Equations (16) and (17) are used to calculate $\:\mathrm{L}{\mathrm{B}}_{\mathrm{i}}\:$and $\:\mathrm{U}{\mathrm{B}}_{\mathrm{i}}\:$. According to Eq. (15), $\:T{st}_{i}$ must exist at this period. This constraint is critical for user satisfaction, as it guarantees that EVs are fully charged by its scheduled departure time^4^.
  15 $$\:\mathrm{L}{\mathrm{B}}_{\mathrm{i}}\le\:T{st}_{i}\le\:\mathrm{U}{\mathrm{B}}_{\mathrm{i}}$$
  16 $$\:\mathrm{L}{\mathrm{B}}_{\mathrm{i}}={T}_{{Arrival}_{\:\mathrm{i}}}$$
  17 $$\:\mathrm{U}{\mathrm{B}}_{\mathrm{i}}={T}_{{Departure}_{\:\mathrm{i}}}{-CD}_{\mathrm{i}}$$

#### Real-time charging schedule approach

This strategy dynamically adjusts the charging schedules of newly arrived electric vehicles (EVs) in each time slot based on real-time grid conditions.

- *Optimization control variables*.

At this optimization stage, the charging start time of the newly arrived EVs is considered as the control variable and is used to construct the charging matrix, as shown in Table 4. In the real-time charging framework, the set of control variables is limited to only the newly arrived EVs at each time slot.

**Table 4:**
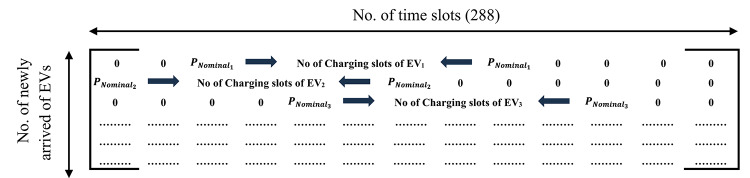
Charging matrix for the newly arrived EVs.

- The multi-objective function consists of:

1. Minimizing the load variance.
  18 $$\:{f}_{1-RT}^{1st}=\sum\:_{t=0}^{T}{({P}_{t}^{Load}+\sum\:_{m=1}^{{N}_{EV"}}{P}_{t,m}^{EV}-{P}_{Avg}^{T})}^{2}$$
  19 $$\:{P}_{Avg}^{T}=\frac{1}{T}*\sum\:_{t=0}^{T}({P}_{t}^{Load}+\sum\:_{m=1}^{{N}_{EV"}}{P}_{t,m}^{EV})$$
  Where $\:{N}_{EV"}$ is the number of newly arrived EVs at time slot t.
2. Minimizing the total charging cost.
  20 $$\:{f}_{2-RT}^{1st}=\sum\:_{i=1}^{{N}_{EV"}}Charging\:Cost$$
3. Minimizing the maximum power.
  21 $$\:{f}_{3-RT}^{1st}={P}_{peak}$$

*Normalization* is performed in accordance with Eq. (10) within the day-ahead charging framework.

The overall objective function is formulated as a weighted sum of the individual objective functions, as defined in Eq. (22). The weighting coefficients (W1 – W3), each set to 0.4, 0.3, and 0.3, respectively.

22 $$\:Multi\:Objective\:Function\:\left(F\right)=\mathrm{W}1\mathrm{*}{f}_{1-RT}^{{\prime\:}{\prime\:}1st}+\mathrm{W}2\mathrm{*}{f}_{2-RT}^{{\prime\:}{\prime\:}1st}+\mathrm{W}3\mathrm{*}{f}_{3-RT}^{{\prime\:}{\prime\:}1st}$$

*Constraints*: The optimization is subject to the state-of-charge limits and the charging start-time constraints, as specified in Eqs. (14) and (15) of the day-ahead charging model.

### Second stage optimization

The primary objective of the second optimization stage is to determine the most effective reactive power dispatch of EVs to regulate voltage levels and minimize power losses. Reactive power dispatch can be addressed in two ways: it can be integrated into the same optimization stage with the optimal charging/discharging schedule, or it can be handled as a separate stage. When integrated, a single optimization process is executed to determine both the EV charging/discharging schedule and the optimal reactive power dispatch simultaneously, as demonstrated in^21^ and^22^. Alternatively, in the two-stage approach, the first stage determines the optimal charging schedule, and its results serve as inputs for the second stage, which focuses exclusively on reactive power dispatch, as demonstrated in^23^. In both approaches, voltage constraints are enforced within ± 0.05 p.u., as reactive power dispatch plays a pivotal role in enhancing voltage stability and ensuring reliable network performance.

In the proposed framework, the reactive power dispatch of EVs is implemented as a separate stage from the optimal charging schedule. The output of the first stage, whether obtained through a day-ahead or real-time approach, serves as the input for the second stage. The second stage operates in real time and is executed independently of whether the first stage adopts a day-ahead or a real-time charging schedule. In this stage, the available EVs are optimized to either inject or absorb reactive power, thereby supporting voltage regulation. Each EV’s reactive power capability is limited to a value determined by its current charging status.

However, determining the optimal reactive power dispatch for all EVs across the entire grid creates a very large and complex search space, because of the huge number of possible combinations of EV contributions at different nodes as mentioned in^23^. To simplify this problem and reduce the associated computational burden, the reactive power dispatch is determined at the node level rather than at the individual EV level. As a result, the number of control variables is significantly reduced. Instead of optimizing the reactive power contribution of each individual EV, the system only needs to manage the aggregated reactive power at each node. Consequently, the number of control variables becomes proportional to the number of network nodes, rather than the number of EVs.

At each time step t, the maximum amount of reactive power that can be injected or absorbed at a given node is determined by the number of EVs connected to that node and their instantaneous charging power levels, as defined in Eq. (23). To ensure customer satisfaction, priority is always given to the active charging power of EVs, while the provision of reactive power is treated as a secondary service constrained by the remaining charger capacity. where n denotes the number of nodes $\:{N}_{evi}$ is the number of EVs connected at node n at time t. $\:{Q}_{max,n,t}$ is the max reactive power can be injected/absorbed by EVs at node n and time slot t. $\:{S}_{{Rated}_{i}}$ is the rated apparent power of EV charger number i and $\:{P}_{{ev}_{i,t}}$ is the charging power of EV number i and time t. Therefore, the node-level reactive power aggregation does not result in overestimation of available reactive power and avoids inverter saturation conflicts, while maintaining computational feasibility.

23 $$\:{Q}_{max,n,t}=\sum\:_{i=1}^{{N}_{evi}}\sqrt{{(S}_{{Rated}_{i}})^2-{(P}_{{ev}_{i,t}})^2}$$

- **Optimization control variables**.

The output from this stage is the reactive power dispatch at each node at the current time slot as shown in Table 5. Where $\:{Q}_{1,t}$ is the optimal reactive power dispatch at node 1 at time slot t.

**Table 5:**
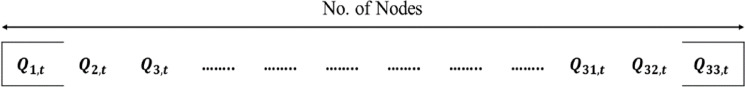
Reactive power dispatch of each node at the current time slot.

- **The multi-objective function consists of: -**.

1. Minimizing the active power losses across the distribution network.
  24 $$\:{f}_{1-RT}^{2nd}=\sum\:_{i=1}^{{N}_{L}}{P}_{Losses}$$
2. Minimizing the reactive power losses across the distribution network.
  25 $$\:{f}_{2-RT}^{2nd}=\sum\:_{i=1}^{{N}_{L}}{Q}_{Losses}$$

*Normalization* is performed according to Eq. (10) within the day-ahead charging framework. The overall objective function is formulated as a weighted sum of the individual objective functions, as expressed in Eq. (26). The weighting factors $\:\mathrm{W}1$ and $\:\mathrm{W}2$ are 0.5 each.

26 $$\:Multi\:Objective\:Fun:\:.\left(F\right),\mathrm{t}=\mathrm{W}1\mathrm{*}{f}_{1-RT}^{{\prime\:}{\prime\:}2nd}+\mathrm{W}2\mathrm{*}{f}_{2-RT}^{{\prime\:}{\prime\:}2nd}$$

- **Constrains**.

1. Voltage
  In this stage, voltage drop is enforced as a constraint across all n feeders, with a tighter permissible limit of 5%.
  27 $$\:\left|{V}_{rated}-{V}_{b}\right|\le\:0.05$$
2. Inverter maximum rated power
  28 $$\:{{Q}_{{\mathrm{I}\mathrm{N}\mathrm{V}}_{i}}}^{2}+{{P}_{{\mathrm{I}\mathrm{N}\mathrm{V}}_{i}}}^{2}\le\:{{S}_{{\mathrm{I}\mathrm{N}\mathrm{V}}_{i}}}^{2}$$

In this expression, $\:{P}_{{\mathrm{I}\mathrm{N}\mathrm{V}}_{i}}$ denotes the active charging power of EV number i assigned to the inverter during the first stage of scheduling. The term $\:{Q}_{{\mathrm{I}\mathrm{N}\mathrm{V}}_{i}}$ refers to the reactive power managed by the inverter of EV number i, which may be either injected into or absorbed from the grid, depending on system requirements. Finally, $\:{S}_{{\mathrm{I}\mathrm{N}\mathrm{V}}_{i}}$ represents the inverter’s maximum rated apparent power of EV number i, defining the upper limit of its combined active and reactive power handling capability.

**Fig. 5:**
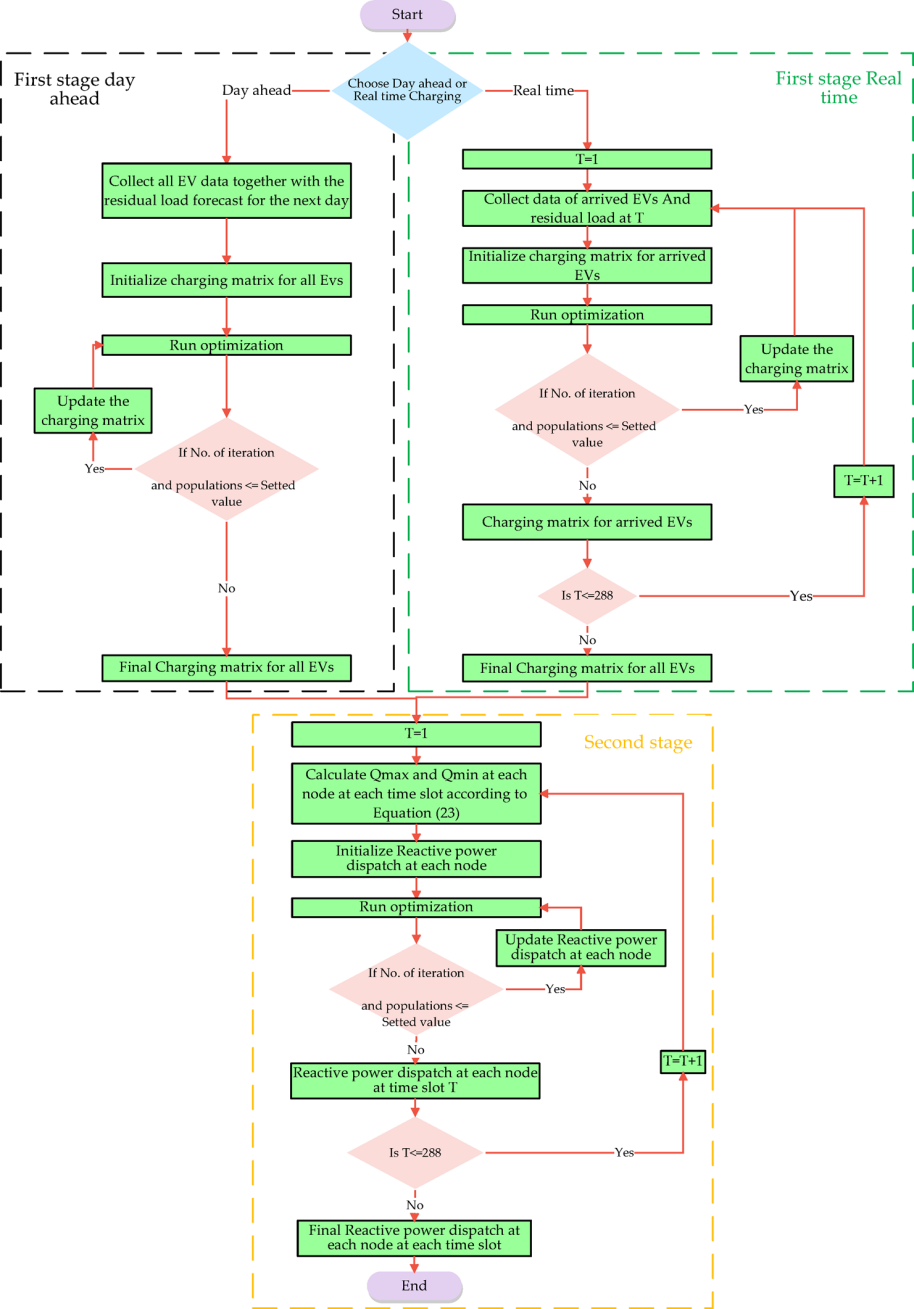
Flow chart of the proposed methodology.

The complete flowchart of the two-stage methodology is presented in Fig. 5. The flowchart illustrates the correct sequence of the proposed methodology, where the EV charging schedule is first determined using either a day-ahead or a real-time approach. The resulting charging schedule is then used as an input to the second stage, which focuses on the reactive power dispatch of EVs.

## Test system and EVs data

### System and line data

**Fig. 6:**
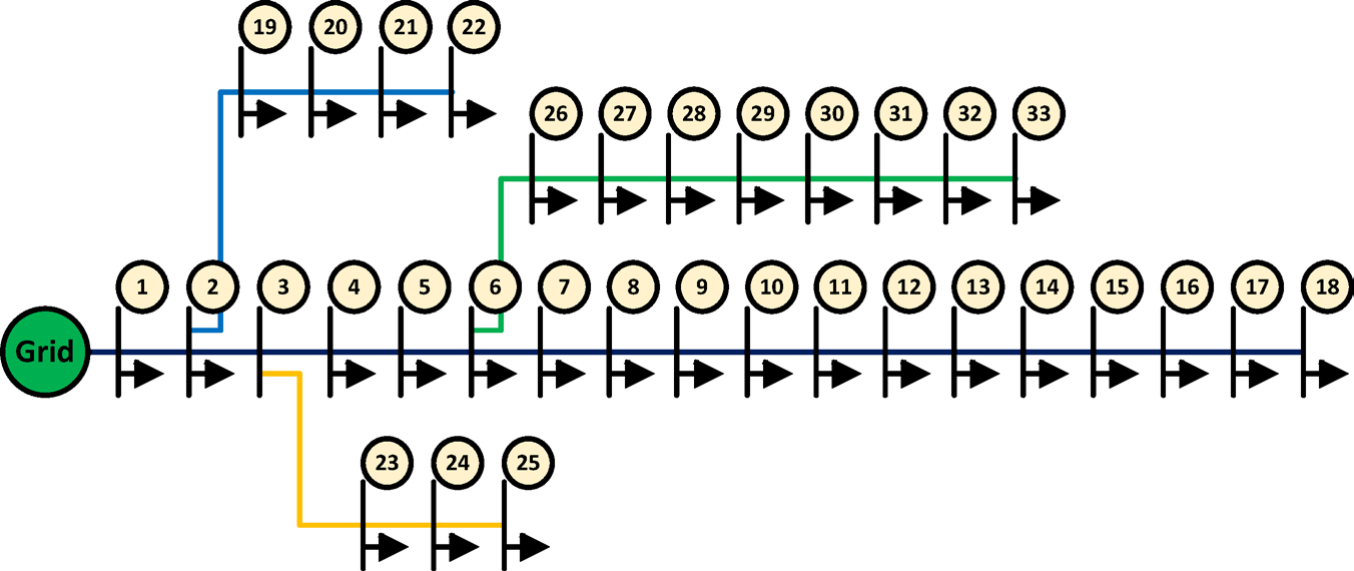
IEEE 33 BUS Radial power distribution.

In this study, the 33-bus radial distribution network, shown in Fig. 6. is selected as the test system to evaluate the performance of the proposed methodology. The network’s technical characteristics, including line impedance data and active/reactive power demands during peak load conditions, are presented in^22^. The system exhibits a peak active load of 3.715 MW without losses, operates at a nominal voltage level of 12.66 kV, and is supported by a substation transformer with a capacity of 5 MVA.

**Fig. 7:**
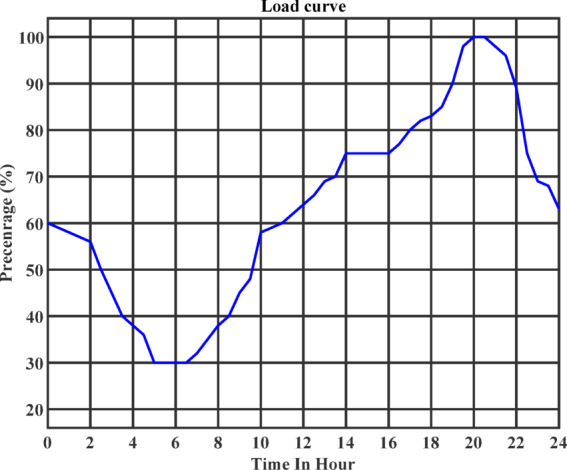
Load profile.

To estimate the load profiles during off-peak hours, the peak active and reactive power values are adjusted using a normalized load percentage profile, as shown in Fig. 7. This profile captures the typical day-ahead variation in consumer demand. It is assumed that all customers have uniform load behavior, adhering to a standardized day-ahead load curve, with maximum demand occurring during the evening hours. The electricity pricing scheme adopted in this study segments the 24-hour day into three distinct periods, off-peak, mid-peak, and peak hours, with the corresponding hourly rates listed in Table 6. These time-of-use (TOU) tariffs are an important factor in shaping consumer behavior and are especially influential in the optimal scheduling of electric vehicles (EVs).

**Table 6:** Time of use tariff.

Hour	Price (USD/kWh)
1–7	0.016
8–17	0.024
18–22	0.03
23–24	0.024

### EVs specifications and user charging preferences

The specifications of EVs considered in this study, including parameters such as charging power, battery capacity, and other relevant characteristics, are provided in Table 7. It should be mentioned that the number of EVs on each bus is presented in Table 8. The arrival and departure times of EV owners are assumed based on the data provided in^22^, which reflects typical day-ahead usage patterns. The initial state of charge (SOC) for each electric vehicle at the time of arrival is set to be a random number between 0.1 and 0.2, representing a relatively low battery level that commonly occurs after a full day of travel or commuting. On the other hand, the desired SOC at the time of departure is set to be a random number between 0.9 and 1, ensuring that the vehicle has enough energy to cover the next day’s travel needs without requiring additional charging. These SOC values serve as boundary conditions in the charging optimization process.

**Table 7:** EV specifications.

Vehicle type	Nissan leaf (EV)
Battery capacity (kWh)	24
Charger capacity (kVA)	6.6
AC charging/discharging power (kW)	3.3
Charging time (h)	7

**Table 8:** No of EVs on each bus.

Bus grouping	Number of EVs	Bus numbers
Group 1	21	5, 6, 9–13, 15–17, 26–28, 33
Group 2	30	2–4, 14, 18–25, 29
Group 3	60	7, 8, 30–32

## Simulation results and discussions

The proposed model is implemented in MATLAB R2020a, utilizing the MATPOWER toolbox for power flow analysis. All simulations are performed on a computer equipped with an Intel Core i7-6500U @ 2.5 GHz processor and 16 GB of RAM. To evaluate the impact of EV parking lots on distribution network performance, six scenarios are considered, reflecting different EV charging strategies and capabilities:

- Reference Scenario: No EV parking lots are considered in the network. This scenario serves as the baseline for comparison.
- Scenario 1 – Unmanaged Charging: EVs are integrated into parking lots with an unmanaged charging strategy, where charging occurs without coordination.

Day-Ahead EVs Charging plan.

- Scenario 2 – Day-Ahead Charging with Active Power Only (Stage 1): This scenario evaluates the effect of day-ahead scheduled EV charging on network performance, considering only active power contributions.
- Scenario 3 – Day-Ahead Charging with Reactive Power Support (Stage 2): In addition to scheduled EVs charging from Scenario 2 (Stage 1), this scenario explores the ability of EVs to inject or absorb reactive power to support voltage regulation.

Real-Time EVs Charging plan.

- Scenario 4 – Real-Time Charging with Active Power Only (Stage 1): This scenario evaluates the effect of real-time scheduled EV charging on network performance, considering only active power contributions.
- Scenario 5 – Real-Time Charging with Reactive Power Support (Stage 2): In addition to scheduled EVs charging from Scenario 4 (Stage 1), this scenario explores the ability of EVs to inject or absorb reactive power to support voltage regulation.

Before presenting the results, it is important to outline the main performance indicators used for comparison across the six EV charging scenarios. The analysis examines how different EV charging strategies affect both the technical and economic aspects of the distribution network. The comparison includes:

- Apparent, active, and reactive power profiles at the primary substation.
- Active and reactive power losses across the distribution network.
- Voltage profiles and stability analysis.
- Charging cost, reflecting the economic efficiency of each charging strategy.

### Metaheuristic algorithm analysis

**Fig. 8:**
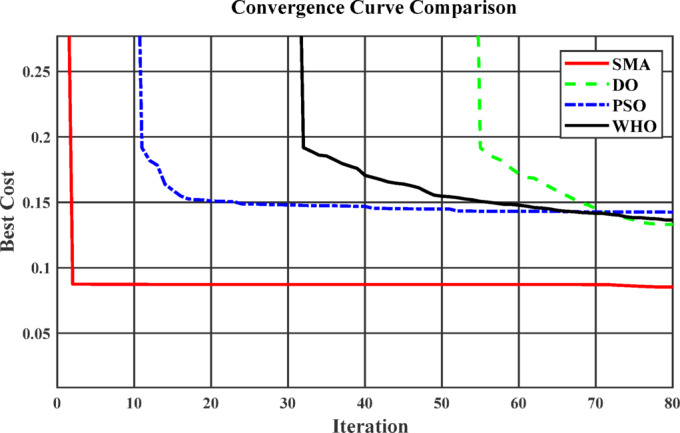
Convergence curve comparison between different optimization methods.

Several metaheuristic optimization algorithms are applied to solve the problem, including Particle Swarm Optimization (PSO), Dandelion Optimization (DO), Wild Horse Optimization (WHO), and Slime Mould Optimization (SMA). Table 9 shows the statistical results obtained from PSO, DO, WHO, and SMA algorithms for scenario 2 where all methods were executed under the same fitness function formulation and subject to identical operational constraints. With regard to Table 9, the SMA algorithm demonstrates outstanding capability in achieving the global optimum solution, attaining the lowest fitness function among all compared methods. The results indicate that SMA effectively balances multiple objectives, simultaneously minimizing the total charging cost and reducing grid related impacts, such as power losses and maximum power demand. This superior performance reflects its strong search ability and adaptive exploration–exploitation mechanism, which enhances its robustness when handling complex nonlinear optimization problems. As shown in Fig. 8, SMA exhibits faster convergence and provides more stable solutions compared to the other algorithms.

**Table 9:** Comparison between different metaheuristic algorithms (Scenario 2).

Aspect	$\:{\mathbf{V}}_{\mathbf{m}\mathbf{i}\mathbf{n}}$(*p*.u)	$\:{\mathbf{P}}_{\mathbf{p}\mathbf{e}\mathbf{a}\mathbf{k}}$(KW)	$\:{\boldsymbol{P}}_{{\boldsymbol{L}\boldsymbol{o}\boldsymbol{s}\boldsymbol{s}\boldsymbol{e}\boldsymbol{s}}_{\boldsymbol{p}\boldsymbol{e}\boldsymbol{a}\boldsymbol{k}}}$(KW)	Total charging cost (USD)
SMA	0.91309	3924.31	202.708	394.1933
PSO	0.909	4150.32	220.82	418.5802
WHO	0.909	4183.93	218.13	417.4846
DO	0.9329	4153.9253	217.8253	411.9186

To demonstrate the robustness and superior performance of the SMA compared with other optimization algorithms, Table 10 summarizes the best, mean, and worst fitness values, as well as the standard deviation (STD), obtained from 30 independent trials of scenario 3 at time slot 245. It can be noted that the best performance was achieved by the SMA.

The Wilcoxon signed-rank test was applied to statistically compare the performance of SMA against PSO, WHO, and DO at a significance level of α = 0.05. This non-parametric test evaluates whether there is a significant difference between two paired sets of results. In the Table 11, p represents the p-value of the test, and h indicates the test decision, where h = 1 means the null hypothesis (i.e., no difference) is rejected, and h = 0 means it is not rejected. Since all reported p-values (9.3157e − 06, 0.0278, and 0.0256) are less than 0.05 and h = 1 in all cases, the results confirm that the SMA performs significantly differently (and better) than PSO, WHO, and DO.

**Table 10:** Performance of metaheuristic algorithms under multiple runs.

Aspect	Best	Worst	Mean	STD
SMA	0.4967	0.5421	0.5141	0.0106
PSO	0.5099	0.5646	0.5302	0.0126
WHO	0.5083	0.543	0.5165	0.0091
DO	0.5041	0.5675	0.5275	0.0147

**Table 11:** Wilcoxon signed-rank test results comparing SMA with PSO, WHO, and DO.

Aspect	*p*	h
SMA vs. PSO	9.3157e−06	1
SMA VS WHO	0.0278	1
SMA VS DO	0.0256	1

The Slime Mould Optimization (SMA), proposed in^38^, is a nature-inspired metaheuristic modeled after the bio-oscillatory foraging behavior of Physarum polycephalum. This organism dynamically constructs optimal pathways to food sources by adaptively balancing exploration and exploitation. SMA simulates this process through a weight-based adaptive mechanism in which candidate solutions are updated according to their fitness ranking, mimicking the contraction and reinforcement of the organism’s venous network toward promising regions.

### Simulation results

#### Apparent, active, and reactive power profiles at the primary substation

**Fig. 9:**
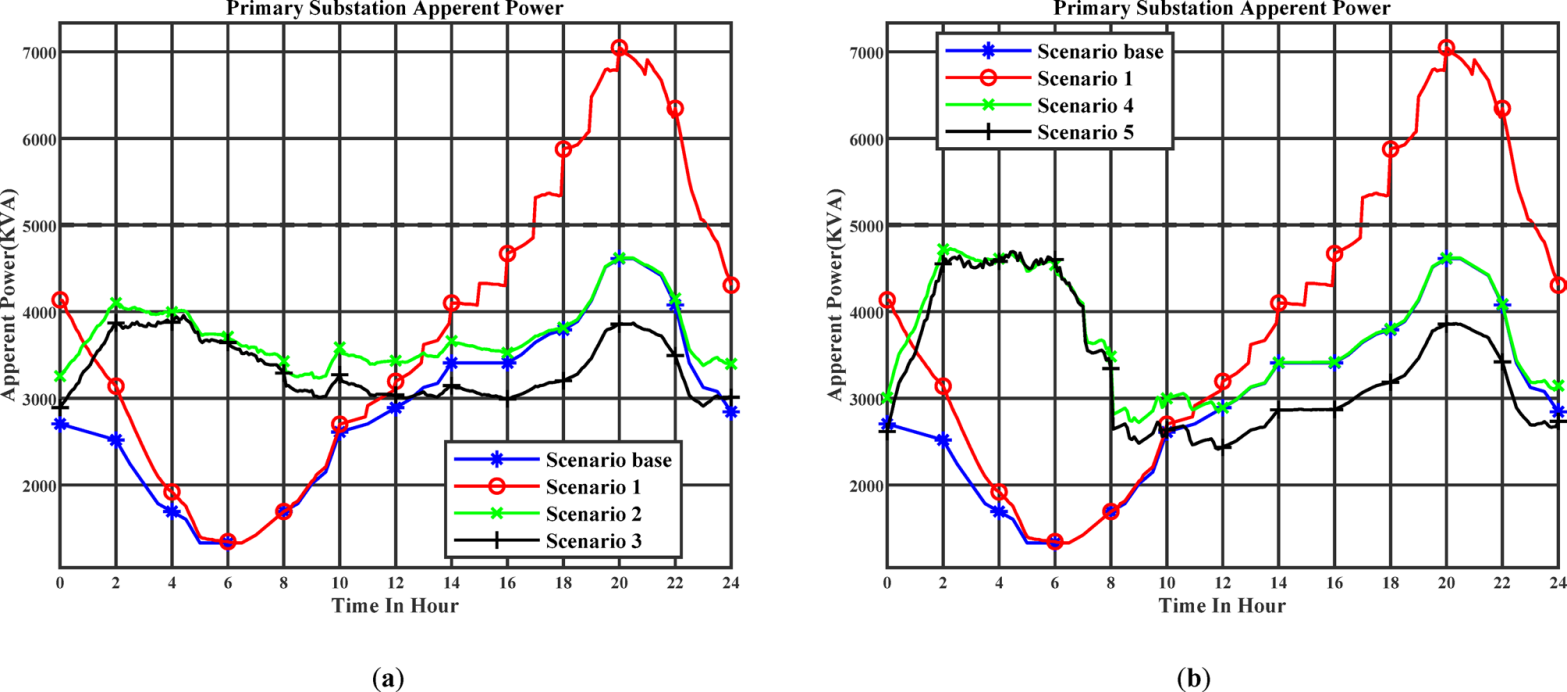
(**a**) The day-ahead apparent power profile of primary substation (Day-ahead charging schedule), (**b**) The day-ahead apparent power profile of primary substation (Realtime charging schedule).

Figure 9 (a) illustrates the apparent power profile at the primary substation throughout the day under the day-ahead planning schedule. Stage 1 corresponds to Scenario 2, while Stage 2 corresponds to Scenario 3. Figure 9 (b) illustrates the apparent power profile at the primary substation throughout the day under the real-time planning schedule. Stage 1 corresponds to Scenario 4, while Stage 2 corresponds to Scenario 5. In Scenario 1, where EV charging is uncoordinated, most vehicles are connected to the grid during the evening peak period between 16:00 and 18:00. This behavior reflects typical user habits, as EV owners tend to plug in their vehicles immediately upon arriving at parking facilities after work. Under this unmanaged charging strategy, the maximum apparent power demand reaches 7.047 MVA, significantly exceeding the substation’s rated capacity of 5 MVA.

In contrast, after applying the first stage of optimization to manage the EV charging schedule in Scenarios 2 and 4, charging is deliberately shifted away from peak demand hours to off-peak periods with lower network loading. This coordinated scheduling effectively reduces the maximum apparent power at the substation. The maximum apparent power is reduced to 4.618 MVA in Scenario 2 and 4.728 MVA in Scenario 4. Scenario 2 achieves a lower maximum apparent power than Scenario 4 because the day-ahead scheduling framework has access to full next-day information, including system conditions and EV availability. The optimization is executed once to generate a coordinated charging plan for the entire next day, enabling the algorithm to obtain a globally optimal solution over the full 24-hour horizon. In contrast, the real-time scheduling strategy in Scenario 4 executes the optimization at every time slot using only the current system data and instantaneous EV availability. These real-time adjustments make the algorithm inherently myopic, causing it to converge to locally optimal results rather than a global optimum. Furthermore, after applying the second stage, where EVs are enabled to inject or absorb reactive power, the maximum apparent power is further reduced to 3.957 MVA in Scenario 3 and 4.693 MVA in Scenario 5. This demonstrates the additional benefit of reactive power coordination in improving network performance and maintaining substation loading within rated limits.

**Fig. 10:**
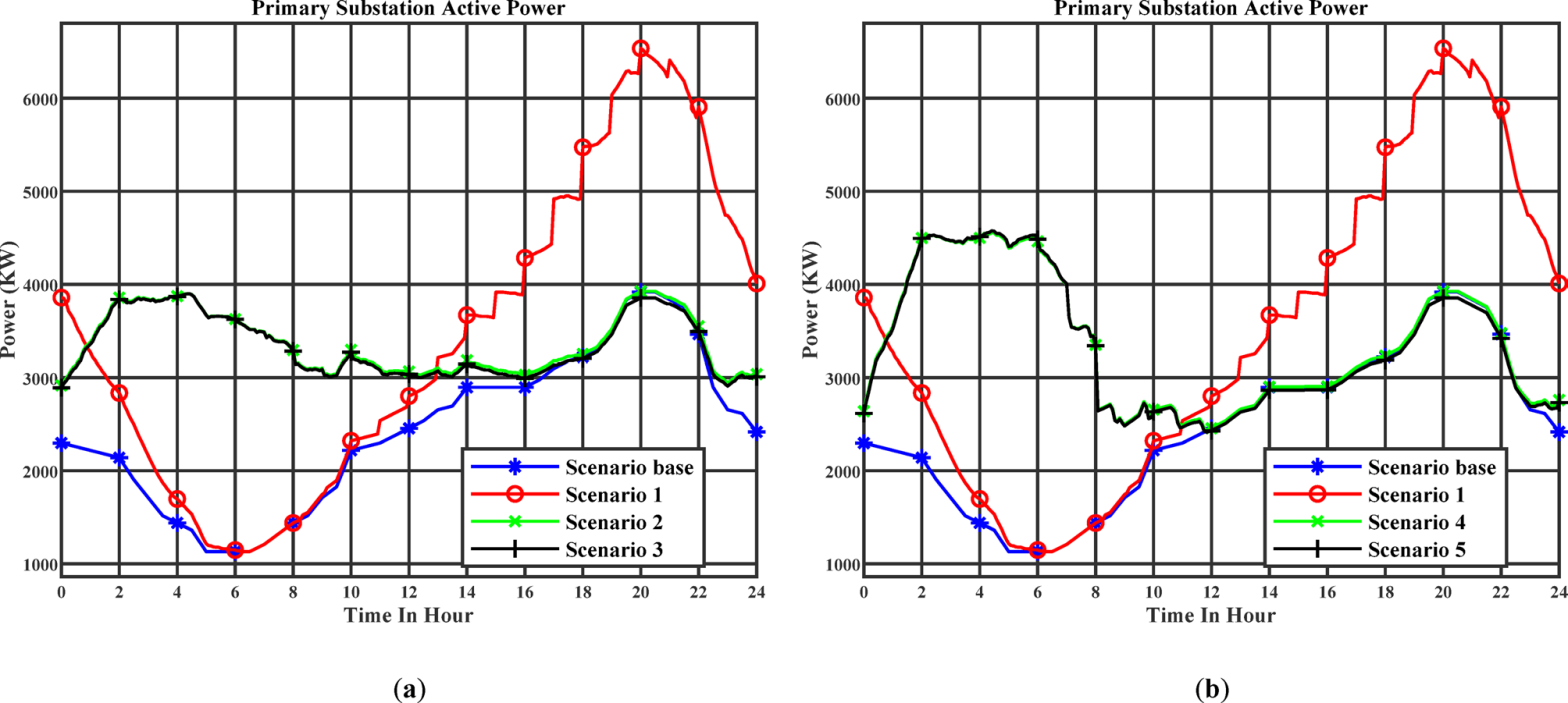
(**a**) The day-ahead active power profile of the primary substation (Day-ahead charging schedule), (**b**) The day-ahead active power profile of the primary substation (Real-time charging schedule).

Figure 10(a) illustrates the active power profile at the primary substation throughout the day under the day-ahead planning schedule. Stage 1 corresponds to Scenario 2, while Stage 2 corresponds to Scenario 3. Figure 10 (b) illustrates the active power profile at the primary substation throughout the day under the real-time planning schedule. Stage 1 corresponds to Scenario 4, while Stage 2 corresponds to Scenario 5. The elevated electricity tariffs are high between 18:00 and 22:00, while the low tariffs occur between 01:00 and 07:00, as shown in Table 6. These higher prices discourage EV charging during peak-tariff periods and incentivize charging during low-tariff hours. As a result, a noticeable increase in active power demand occurs between 01:00 and 07:00, when electricity tariffs are more favorable, making this window a more economical time for charging. Coordinated charging cause flatten load curve, especially in the day-ahead charging schedule.

**Fig. 11:**
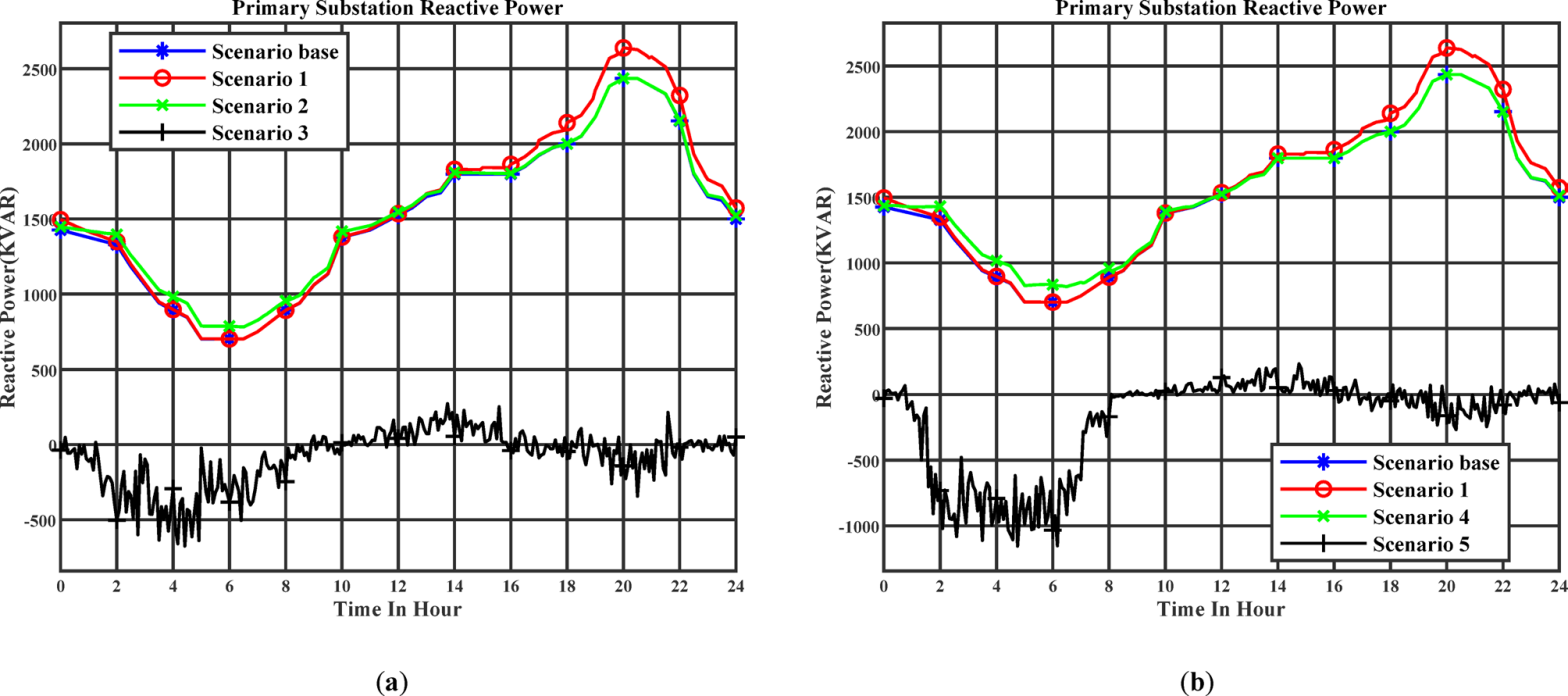
(**a**) The day-ahead reactive power profile of the primary substation (Day-ahead charging schedule), (**b**) The day-ahead reactive power profile of the primary substation (Real-time charging schedule).

Figure 11 (a) illustrates the reactive power profile at the primary substation throughout the day under the day-ahead planning schedule. Stage 1 corresponds to Scenario 2, while Stage 2 corresponds to Scenario 3. Figure 11 (b) illustrates the reactive power profile at the primary substation throughout the day under the real-time planning schedule. Stage 1 corresponds to Scenario 4, while Stage 2 corresponds to Scenario 5. The reactive power at the primary substation tends to approach zero, especially in Scenario 3. By compensating the reactive power demand, this capability effectively minimizes the reactive power flow through the substation, thereby reducing the overall apparent power burden on the system, minimizing both active and reactive power losses, and improving the voltage profile throughout the day. It can also be observed that from 02:00 AM to 6:00 AM, more reactive power is injected since the active power demand increases during this period due to the larger number of EVs being charged, driven by the lower charging cost.

#### Active and reactive power losses across the distribution network

**Fig. 12:**
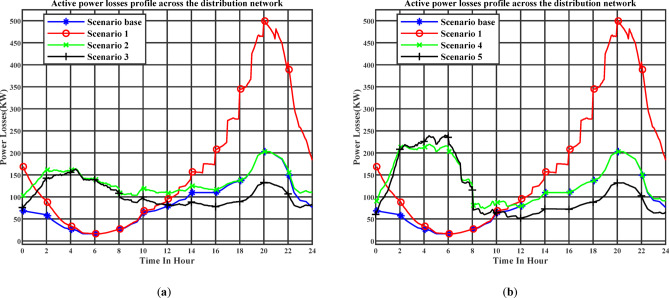
(**a**) The day-ahead active power losses profile across the distribution network (Day-ahead charging schedule), (**b**) The day-ahead active power losses profile across the distribution network (Realtime charging schedule).

Figure 12 (a) illustrates the day-ahead active power losses profile across the distribution network throughout the day under the day-ahead planning schedule. Stage 1 corresponds to Scenario 2, while Stage 2 corresponds to Scenario 3. Figure 12 (b) illustrates the day-ahead active power losses profile across the distribution network throughout the day under the real-time planning schedule. Stage 1 corresponds to Scenario 4, while Stage 2 corresponds to Scenario 5. In Scenarios 3 and 5, where electric vehicles are allowed to provide reactive power support, a noticeable improvement in power-loss reduction is achieved. The maximum active power loss decreases to 163.23 kW with 2.552 MWh of daily energy losses in Scenario 3, and to 240 kW with 2.771 MWh in Scenario 5, compared with 500.61 kW and 4.04 MWh in the uncoordinated charging case (Scenario 1).

**Fig. 13:**
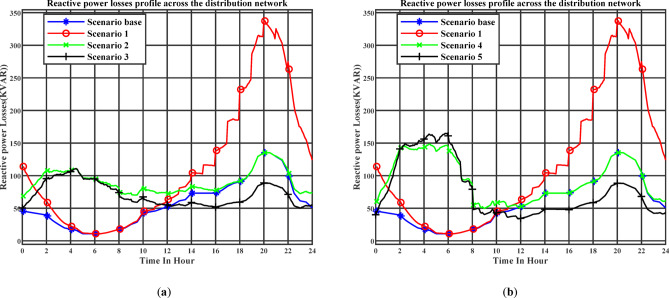
(**a**) The day-ahead reactive power losses profile across the distribution network (Day-ahead charging schedule), (**b**) The day-ahead reactive power losses profile across the distribution network (Realtime charging schedule).

Figure 13 (a) illustrates the day-ahead reactive power losses profile across the distribution network throughout the day under the day-ahead planning schedule. Stage 1 corresponds to Scenario 2, while Stage 2 corresponds to Scenario 3. Figure 13 (b) illustrates the day-ahead reactive power losses profile across the distribution network throughout the day under the real-time planning schedule. Stage 1 corresponds to Scenario 4, while Stage 2 corresponds to Scenario 5. The maximum reactive power loss decreases to 110.84 kVar in Scenario 3 and to 164.9 kVar in Scenario 5, compared with 338.09 kVar in the uncoordinated charging case (Scenario 1).

In scenario 5 in Figs. 11 and 13, both reactive power and reactive power losses exhibit a noticeable increase during the early morning period between 01:00 and 07:00. This is attributed to the higher number of EVs charging during this low-tariff period, which causes a voltage drop. Consequently, greater reactive power injection by the chargers is required to compensate for the voltage decrease. The main objective of this reactive support is to maintain the voltage above the minimum threshold of 0.95 p.u.

#### Voltage profile and stability analysis

**Fig. 14:**
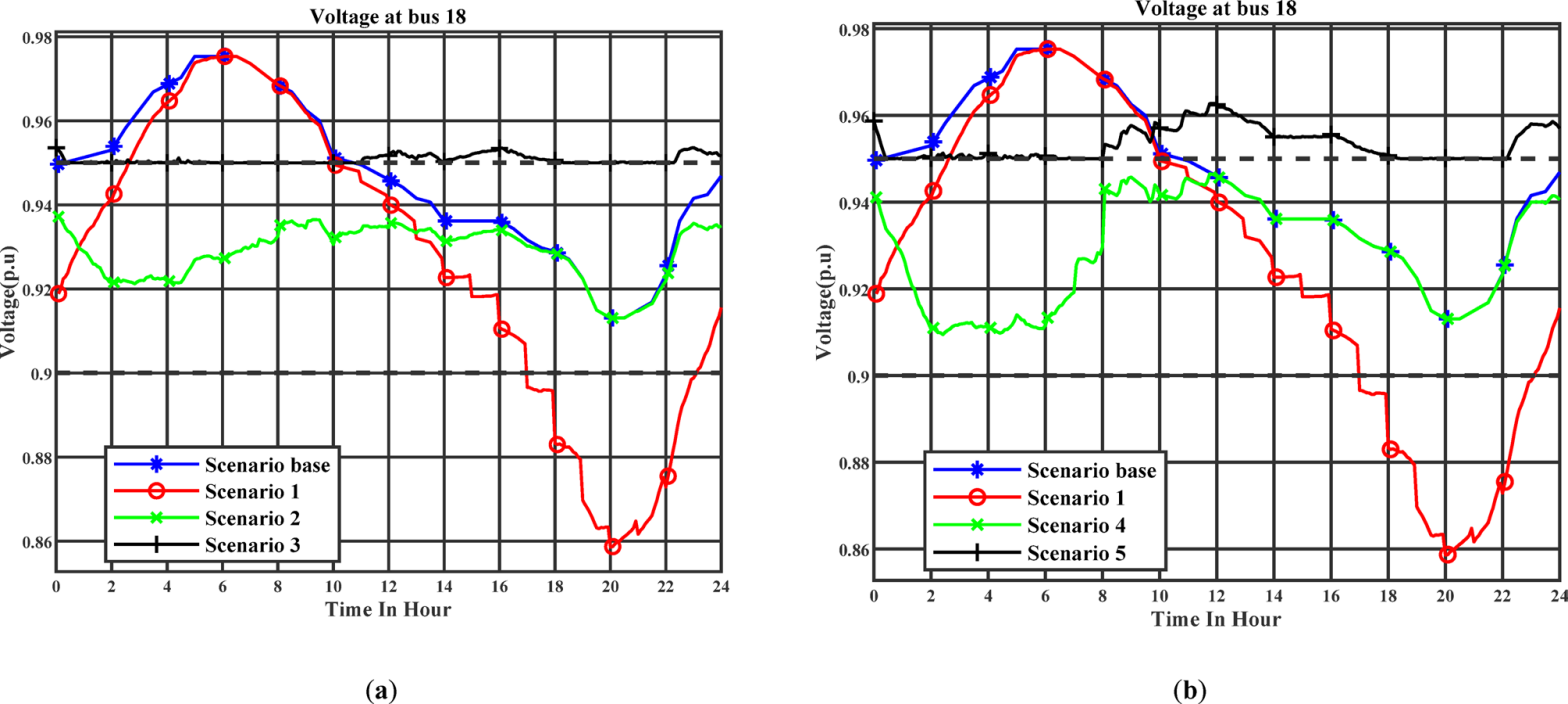
(**a**) The voltage profile at bus 18 (Day-ahead charging schedule), (b) the voltage profile at bus 18 (Real-time charging schedule).

Figure 14 (a) illustrates the day-ahead voltage profile at Bus 18 throughout the day under the day-ahead planning schedule. Stage 1 corresponds to Scenario 2, while Stage 2 corresponds to Scenario 3. Figure 14 (b) illustrates the day-ahead voltage profile at Bus 18 throughout the day under the real-time planning schedule. Stage 1 corresponds to Scenario 4, while Stage 2 corresponds to Scenario 5. Bus 18 is strategically selected as it is the most remote bus from the main supply point and therefore typically experiences the most pronounced voltage drops. In the first optimization stage, voltage deviations are allowed within ± 10% of nominal, while in the second stage, a stricter threshold of ± 5% is enforced. The simulation results demonstrate that under Scenarios 3 and 5, where EV chargers are equipped with the capability to inject or absorb reactive power and are actively managed to support the grid, voltage at Bus 18 remains consistently above the minimum limit of 0.95 p.u. throughout the day. In contrast, Scenario 1, which involves uncoordinated charging, shows a substantial voltage depression, with Bus 18 voltage falling below 0.9 p.u. during peak demand periods. This severe undervoltage condition highlights the risk of allowing unmanaged EV charging in weak parts of the distribution system. Similarly, Scenarios 2 and 4, which implement managed charging but utilize chargers that lack reactive power capabilities, also experience voltage violations, with Bus 18 frequently dropping below the 0.95 p.u. threshold for several consecutive hours. This finding emphasizes that load shifting alone is insufficient for maintaining voltage levels in distant buses if reactive support is not incorporated. It is also worth noting that even in the base scenario (i.e., without the addition of EVs loads), Bus 18 voltage marginally breaches the 0.95 p.u. limit, indicating an already stressed voltage condition in the network. The introduction of reactive power-enabled EV charging substantially improves this situation.

The load-flow analysis converged successfully within three to four iterations across all scenarios, indicating stable numerical performance. The condition number of the reduced Jacobian matrix was computed to further assess numerical stability. It is defined as the ratio between the maximum and minimum singular values, as given in (29), and reflects the sensitivity of the system to small perturbations. A lower condition number indicates better numerical conditioning, whereas increasing values reflect higher sensitivity of the Jacobian matrix and potential proximity to ill-conditioned operating points.

29 $$\:\:Conditioning\:Number\:=\:\:\frac{\delta\:max}{\delta\:min}$$

Table 12 presents the minimum singular value and condition number under different scenarios, confirming that the system remains stable and sufficiently distant from singularity. In particular, Scenario 3 achieves the highest minimum singular value δmin (0.153), and the lowest condition number (1835), indicating improved voltage stability and enhanced numerical conditioning.

**Table 12:** Minimum singular value and Jacobian conditioning under different operating scenarios.

Title	Scenario base	Scenario1	Scenario2	Scenario3	Scenario4	Scenario5
$\:\delta\:min$	0.1453	0.1289	0.1453	0.1531	0.14	0.142
Conditioning number	1932	2173	1934	1835	1974	1887

#### Charging cost reduction

**Fig. 15:**
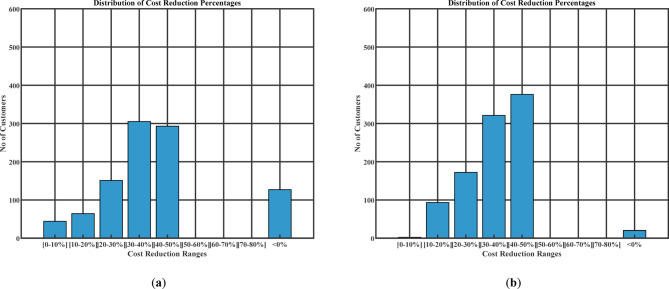
(**a**) Cost reduction percentage (Day-ahead charging schedule), (**b**) cost reduction percentage (Real-time charging schedule).

Figure 15 (a) illustrates the cost reduction for the day-ahead planning schedule. Figure 15 (b) illustrates the cost reduction for the real-time planning schedule. The results show significant cost reduction for most users, while only a few users experience a cost increase greater than the uncoordinated charging. The total charging cost decreases from 552.31 USD in the uncoordinated charging case to 394.19 USD under the Day-Ahead Planning strategy and 363.68 USD with Real-Time Planning.

Table 13 presents a comprehensive summary of the results obtained under various simulation scenarios. Coordinated charging in Scenarios 2 and 4 significantly enhances system performance. The total daily energy losses decrease from 4.04 MWh in Scenario 1 to 3.271 MWh and 3.384 MWh, corresponding to reductions of 19.03% and 16.24%, respectively. In addition, peak demand is substantially reduced, and total charging costs decrease by 28.63% (Scenario 2) and 34.15% (Scenario 4), demonstrating both technical and economic benefits of coordinated scheduling.

When reactive power support is activated (Scenarios 3 and 5), further improvements are achieved. Daily energy losses are reduced to 2.552 MWh and 2.771 MWh, representing total reductions of 36.8% and 31.4%, respectively, compared to the uncoordinated case. Moreover, the minimum bus voltage is restored to 0.95 p.u., satisfying the operational limits (0.95–1.05 p.u.).

**Table 13:** Complete comparison between different scenarios.

Title	Scenario base	Scenario1	Scenario2	Scenario3	Scenario4	Scenario5
$\:{P}_{{Losses}_{peak}}$ (KW)	202.68	500.61	202.71	163.23	219.39	240.06
$\:{E}_{P\_Losses}$(MWh)	2.08	4.04	3.271	2.552	3.384	2.771
$\:{Q}_{{Losses}_{peak}}$ *(KVar)*	135.14	338.09	135.16	110.84	148.24	164.92
$\:{P}_{peak}$ (KW)	3917.68	6535.51	3924.31	3898.91	4555.67	4575.07
$\:{V}_{min}$ (p.u)	0.9131	0.859	0.913	0.95	0.909	0.95
$\:{V}_{max}$ (p.u)	1	1	1	1	1	1
$\:{S}_{peak}$ (MVA)	4612.82	7047.87	4618.46	3957.02	4728.17	4693.51
Total charging cost (USD)	–	552.31	394.1933	–	363.682	–

### Mitigating overvoltage with EVs

**Fig. 16:**
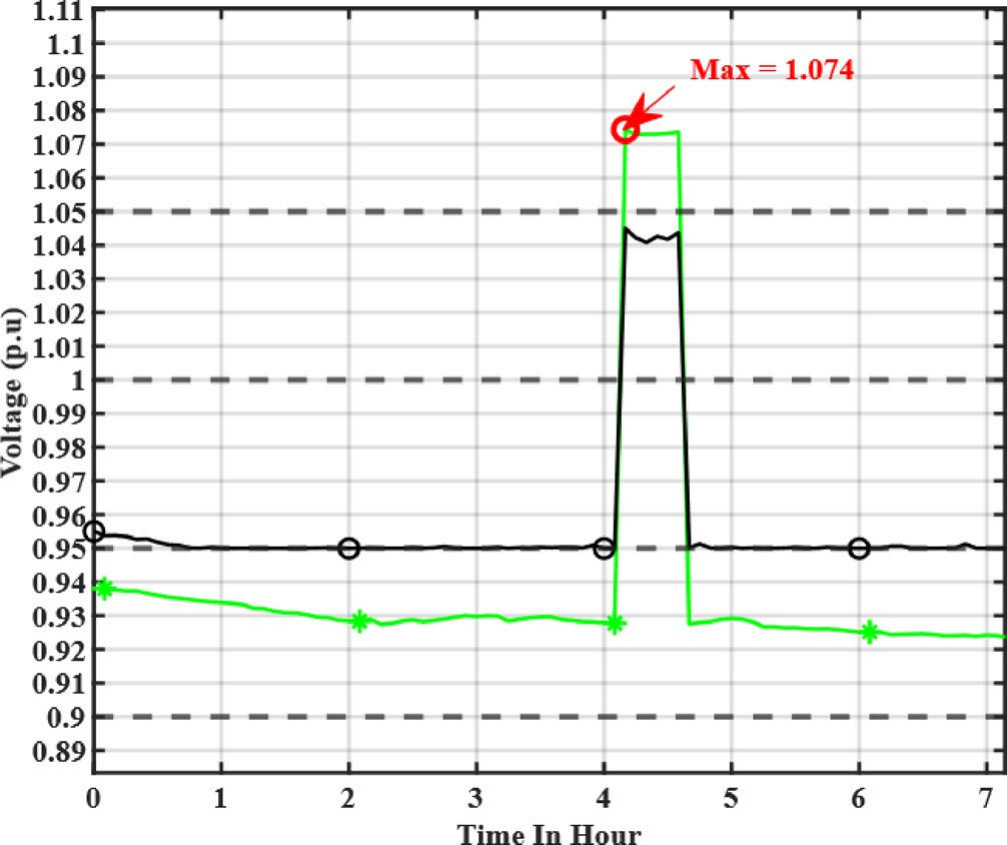
Sudden voltage rise at Bus 18.

A simulation was conducted to evaluate the performance of the proposed algorithm under challenging conditions, such as a sudden cut in load demand. These conditions are known to cause significant overvoltage in distribution networks. In response, the electric vehicles (EVs) were configured to absorb reactive power from the system, which helps reduce the voltage magnitude and bring it back within the standard operating range, below 1.05 per unit, as illustrated in Fig. 16 The voltage at bus 18 rises to 1.074 p.u. following the overvoltage event; however, after applying the proposed reactive power optimization strategy, the voltage is successfully regulated back below 1.05 p.u. This result demonstrates the ability of the proposed algorithm to dynamically adjust EV reactive power behavior and effectively mitigate voltage violations, under varying operating conditions.

### Examining different penetration levels

**Fig. 17:**
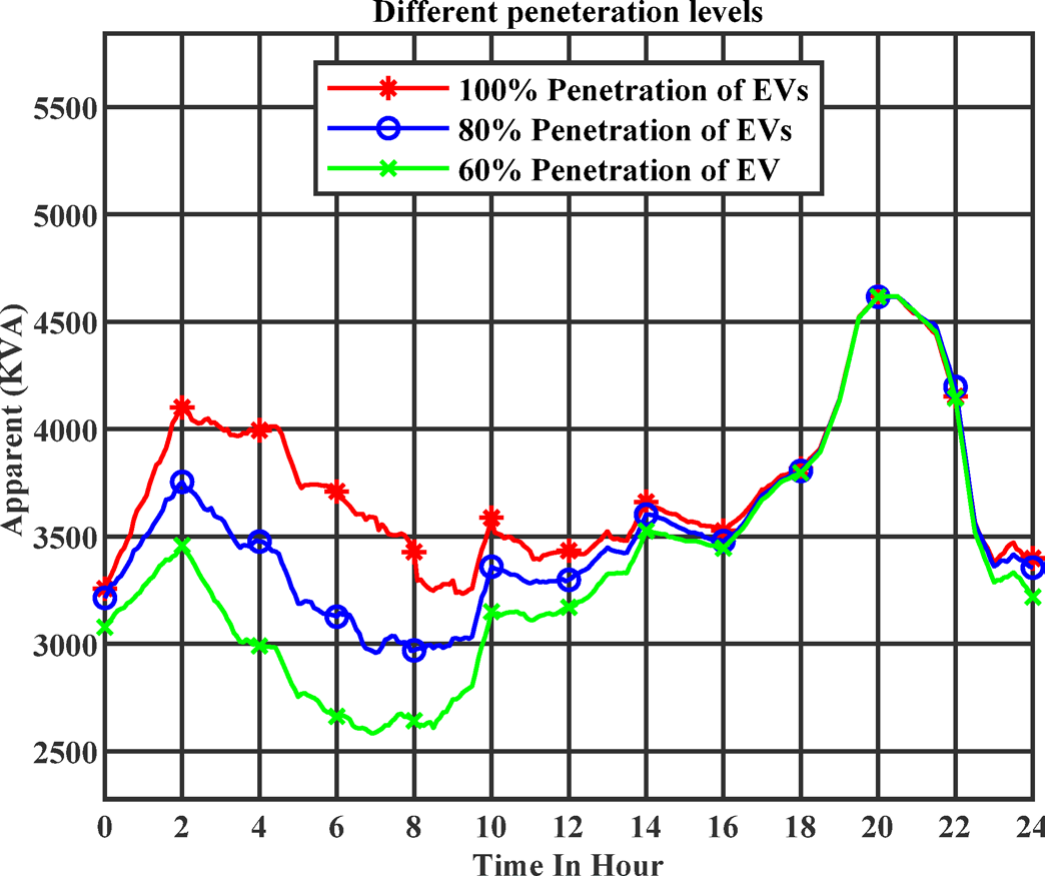
Apparent power profile at 60%, 80%, and 100% EV penetration.

To evaluate the impact of electric vehicle (EV) integration on the distribution network, the charging demand was analyzed under three different penetration levels: 60%, 80%, and 100%. Figure 17 presents the apparent power demand over a 24-hour period for each scenario. The results clearly demonstrate a direct relationship between the level of EV penetration and the system load.

### Examining different priorities of objectives through pareto-optimal solutions

To investigate the trade-offs among the considered objectives, the obtained Pareto-optimal solutions are analyzed under different priority scenarios. In the day-ahead charging framework, five objective functions are initially considered, namely: maximum power losses, voltage deviation, voltage stability index, total charging cost, and load variance. To simplify the multi-objective analysis and improve result interpretability, the five objectives are aggregated into three main performance categories. Specifically, maximum power losses, voltage deviation, and voltage stability index are combined into a single composite metric referred to as system indices, while total charging cost and load variance are maintained as independent objectives. Accordingly, the final Pareto optimization problem is reformulated as a three-objective problem consisting of system indices, total charging cost, and load variance.

**Fig. 18:**
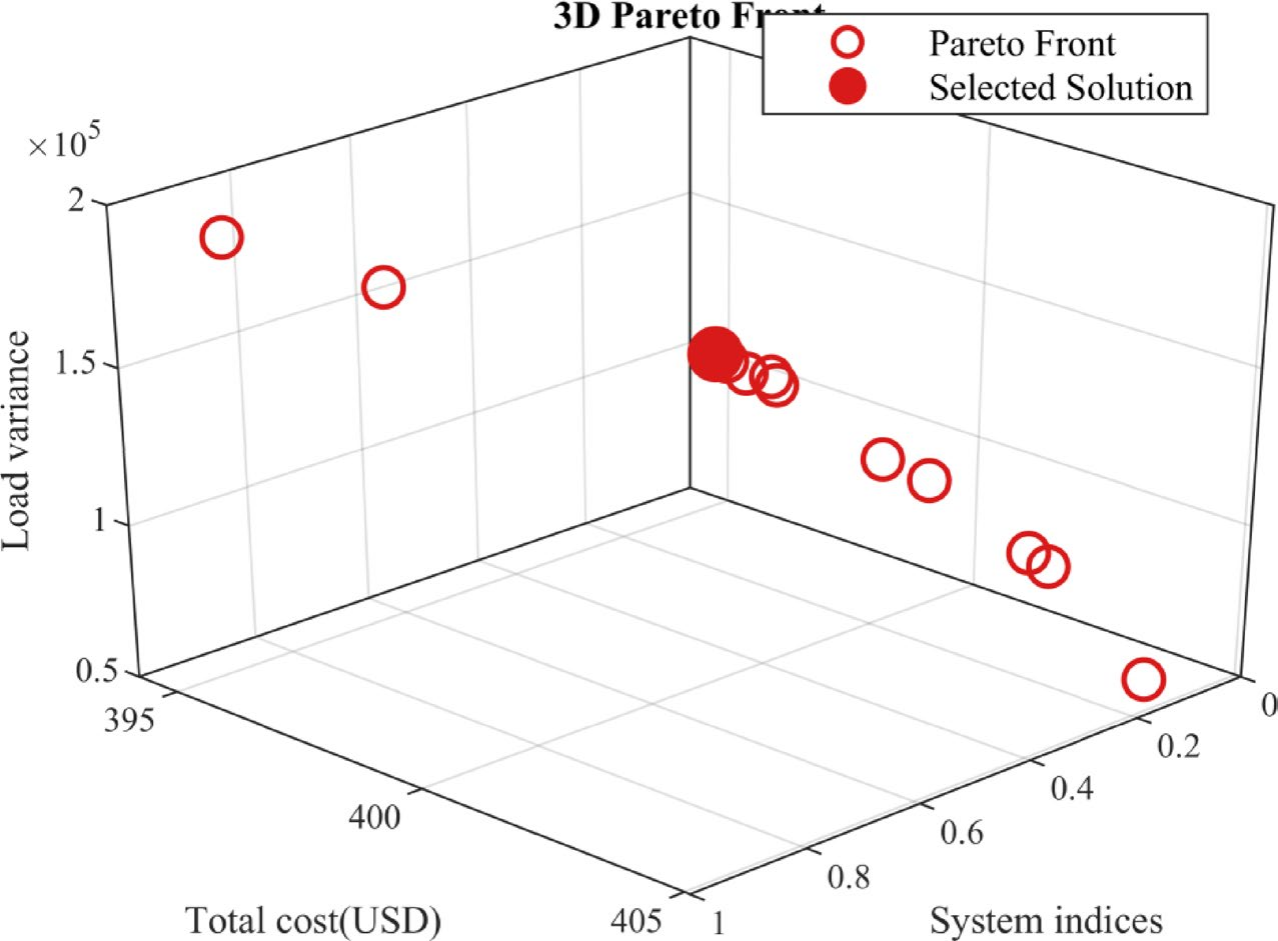
3D Pareto front for system indices, total charging cost, and load variance.

As illustrated in Fig. 18, The resulting Pareto front highlights the inherent trade-offs among the three objectives while clearly separating dominated solutions from the Pareto-optimal set. This provides system operators with the flexibility to select an operating point that best satisfies practical economic, operational, and security requirements.

### Examining uncertainties in EV behavior in real-time charging planning

The day-ahead charging plan is developed based on deterministic forecasted data for all EVs and is implemented in an offline manner, which limits its capability to handle uncertainties in EV behavior. In contrast, the real-time charging strategy operates online by continuously incorporating newly arrived EVs at each time slot, thereby enabling effective adaptation to uncertainty. The uncertainty of EVs behavior arises from random variations in arrival and departure times, initial state of charge, and required energy, which cannot be accurately predicted due to factors such as traffic congestion, unexpected changes in users’ schedules, varying daily travel distances, and different charging preferences.

Accordingly, uncertainty scenarios associated with EV arrival and departure times are generated using the segmented normal distribution functions defined in Eqs. (30) and (31). The shape of the PDFs^21^ is defined by the following values $\:{\mu\:}_{r}$ = 8.92, $\:{\sigma\:}_{r}$ = 3.24, $\:{\mu\:}_{e}$ = 17.47, $\:{\sigma\:}_{e}$ = 3.41. The initial SOC of each EV upon arrival is randomly selected between 0.1 and 0.2, whereas the desired SOC at departure is randomly assigned between 0.9 and 1.0.

30 $$\:{f}_{e\left({t}_{arr}^{m}\right)}=\left\{\begin{array}{c}\frac{1}{\sqrt{2\pi\:}{\sigma\:}_{e}}*\:exp\left(\:-\frac{\left(\:{\left({t}_{arr}^{m}+\:24\:-\:{\mu\:}_{e}\right)}^{2}\right)}{2\:{\sigma\:}_{e}^{2}}\right),\:\:\:0\:<\:t\:\le\:\:{\mu\:}_{e}-\:12\\\:\frac{1}{\sqrt{2\pi\:}{\sigma\:}_{e}}*\:exp\left(\:-\frac{\left(\:{\left({t}_{arr}^{m}-\:{\mu\:}_{e}\right)}^{2}\right)}{2\:{\sigma\:}_{e}^{2}}\right),\:\:\:\:\:\:{\mu\:}_{e}-\:12\:<\:t\:\le\:\:24\end{array}\right.$$

31 $$\:{f}_{r\left({t}_{dep}^{m}\right)}=\left\{\begin{array}{c}\frac{1}{\sqrt{2\pi\:}{\sigma\:}_{r}}*\:exp\left(\:-\frac{\left(\:{\left({t}_{dep}^{m}-\:{\mu\:}_{r}\right)}^{2}\right)}{2\:{\sigma\:}_{r}^{2}}\right),\:\:\:0\:<\:t\:\le\:\:{\mu\:}_{r}+\:12\\\:\frac{1}{\sqrt{2\pi\:}{\sigma\:}_{r}}*\:exp\left(\:-\frac{\left(\:{\left({t}_{dep}^{m}-\:24\:-\:{\mu\:}_{r}\right)}^{2}\right)}{2\:{\sigma\:}_{r}^{2}}\right),\:\:\:{\mu\:}_{r}+\:12\:<\:t\:\le\:\:24\end{array}\right.$$

**Fig. 19:**
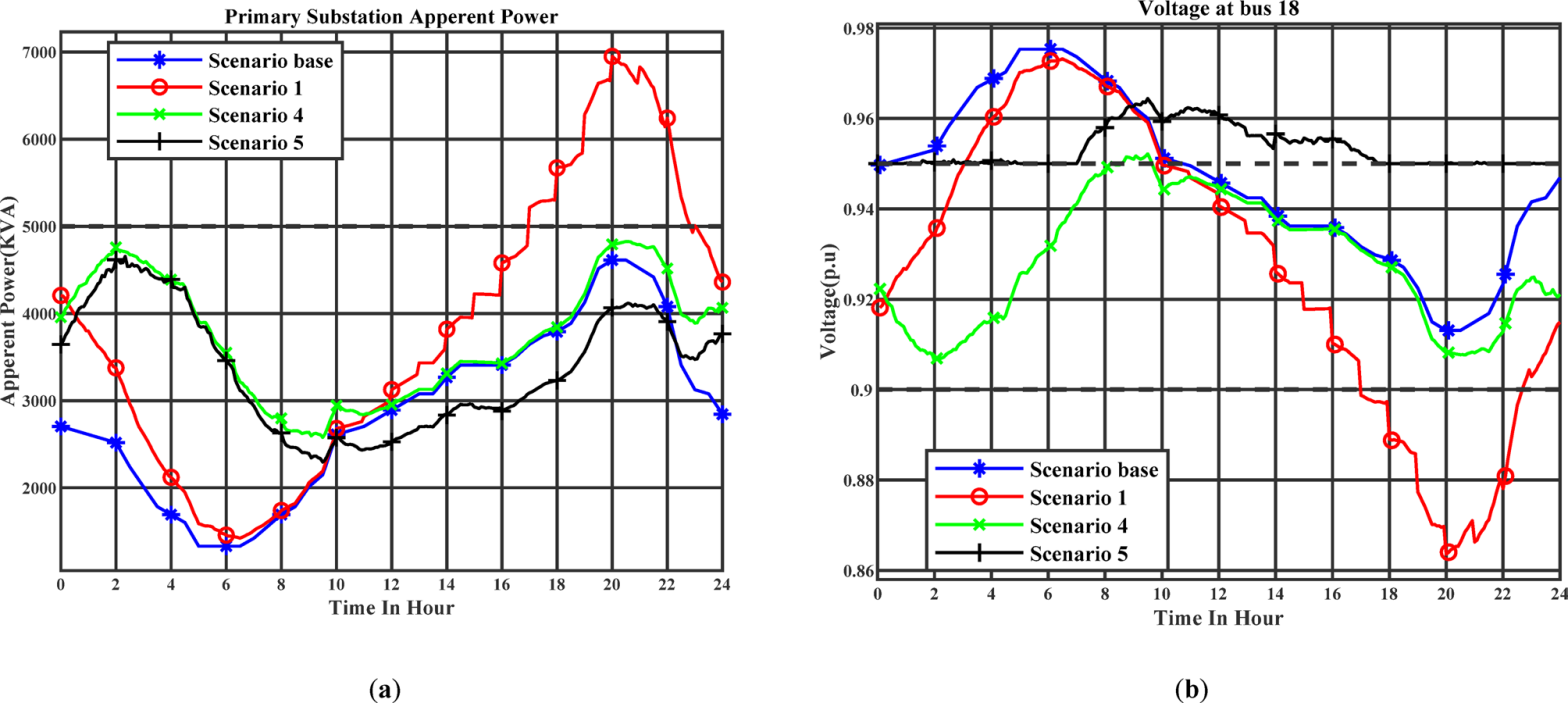
System apparent power and voltage performance under EV behavioral uncertainty in real-time charging planning.

As illustrated in Fig. 19 (a) and (b), both the apparent power and voltage profiles remain within the permissible operational limits despite uncertainty in EV arrival, departure times, and SOC values. This demonstrates the effectiveness of the proposed real-time charging strategy combined with reactive power dispatch in maintaining system stability and operational security under stochastic EV behavior.

### Model complexity and scalability analysis

The computational complexity of the proposed framework is evaluated using Big-O notation^17^ in order to assess its scalability with increasing network size and time-slot resolution. The power flow analysis is performed using the Newton–Raphson method, which has a computational complexity of O ($\:{{N}_{b}}^{1.5}$), where it depends on the number of buses $\:{N}_{b}\:$. The SMA optimization algorithm employs $\:it$ iterations and a population size $\:k$. The population size increases as the number of EVs increases. This adjustment ensures sufficient search capability and improves the likelihood of obtaining the optimal solution as the problem dimension grows.

In the day-ahead charging plan, power flow analysis is carried out at each time slot, over a scheduling horizon consisting of T time intervals at each population $\:k$ and iteration $\:it$. Therefore, the overall computational complexity of the day-ahead planning stage is O ($\:{{it.\:\:k.\:\:T.\:\:N}_{b}}^{1.5}$). This stage has high computational complexity, but it is working offline. Therefore, computational time does not represent a critical constraint in this phase.

In the real-time charging plan, the power flow analysis is not embedded within the optimization loop. Instead, mathematical calculations are performed during optimization, and the load flow is executed only once after the optimization is completed at the current time slot to evaluate network performance. Hence, the computational complexity becomes O ($\:it.k.T+{{.N}_{b}}^{1.5}$).

In the real-time reactive power dispatch, power flow analysis is carried out at each population $\:k$ and iteration $\:it$,,so the computational complexity of O ($\:{{N}_{b}}^{1.5}$). So computational complexity is O ($\:{{it.\:\:k.\:\:N}_{b}}^{1.5}$).

From previous computational complexity, which explains the relatively moderate computational burden is almost linear, especially for real-time charging plan and reactive power dispatch, makes it applicable for online and large-scale implementation.

## Conclusion

This study presents a comprehensive two-stage multi-objective optimization framework designed to address the challenges associated with large-scale electric vehicle (EV) charging patterns. In the first stage, two types of EV charging schedules were developed and compared: day-ahead charging and real-time charging. Day-ahead charging involves planning EV charging over a 24-hour horizon, while real-time charging dynamically adjusts charging behavior based on immediate grid conditions. The second stage leveraged the reactive power capabilities of EV inverters to further reduce both active and reactive power losses, showcasing the potential of EVs as distributed energy resources for grid support. The proposed approach was validated through simulations on a 33-bus radial distribution system, demonstrating its effectiveness in enhancing grid performance and ensuring user satisfaction. Key results of the proposed framework include:

- A significant reduction in the total daily active power losses is achieved under both the Day-Ahead and Real-Time planning strategies compared to the uncoordinated charging scenario. The losses decrease from 4.04 MWh to 2.55 MWh and 2.77 MWh under the Day-Ahead and Real-Time strategies, corresponding to reductions of 36.8% and 31.4%, respectively.
- Voltage profiles are consistently maintained within the acceptable limits of ± 5%.
- In addition, both coordinated strategies result in substantial reductions in EV charging costs compared to the uncoordinated case. The total charging cost decreases from 552.31 USD to 39.19 USD and 363.68 USD under the Day-Ahead and Real-Time strategies, achieving cost savings of 28.63% and 34.15%, respectively.

Different optimization algorithms are implemented to efficiently solve the proposed models, including particle swarm optimization, dandelion optimization, wild horse optimization, and slime mould optimization. Among these techniques, SMA demonstrated superior performance in achieving the optimization goals.

Limitations of the proposed methodology:

- The model assumes that all EV chargers are capable of providing reactive power support.
- Unplanned EV departure events are not considered.
- V2G discharging operation is not included.
- Intra-node reactive power coordination among EVs is not addressed.

Future research directions could explore the integration of renewable energy sources with EV charging infrastructure, the impact of vehicle-to-grid (V2G) technologies on battery degradation, and the scalability of the proposed framework for larger and more complex distribution networks.

## Data Availability

All data generated or analysed during this study are included in this published article.

## List of symbols

EVs: Electric vehicles
V2G: Vehicle to grid
G2V: Grid to vehicle
it: Number of iterations
k: Number of populations
$\:{SOC}_{{req}_{i}}$: Required state of charge for EV number i
$\:{SOC}_{{in}_{i}}$: Initial state of charge for EV number i
$\:{SOC}_{{ev}_{i,t}}$: Current state of charge for EV number i at time t
$$\:\eta\:$$: Charger efficiency
$\:{CE}_{i}$: Charging energy of EV number i
$\:{CD}_{i}$: Charging duration of EV number i
$\:{E}_{i}$: Battery energy of EV number i
$\:{P}_{{Nominal}_{i}}$: Charger power of EV number i
$\:{N}_{EV}$: Number of EVs
$\:{N}_{b}$: Number of nodes
$\:{N}_{L}$: Number of lines
$$\:{V}_{rated}$$: Rated voltage
$$\:{V}_{b}$$: Node voltage
$\:\delta\:min$: The minimum singular value of the Jacobian matrix
$\:{P}_{{Losses}_{peak}}$: Maximum active power losses
$\:{E}_{P\_Losses}$: Daily energy active power losses
$\:{S}_{transformer}$: Transformer rated apparent power
$\:L{B}_{i}$: Lower bound of the starting time of the charge of EV number i
$\:T{st}_{i}$: The starting time of the charge of EV number i
$\:U{B}_{i}$: Upper bound of the starting time of the charge of EV number i
$\:{T}_{{Arrival}_{\:i}}$: Arrival time of EV number i
$\:{T}_{{Departure}_{\:i}}$: Departure time of EV number i
$\:{Q}_{max,n,t}$: Max reactive power can be injected /absorbed by EVs at node n and time slot t
$\:{N}_{evi}$: Number of newly arrived EVs
$\:{S}_{{Rated}_{i}}$: The rated apparent power of EV number i
$\:{P}_{{ev}_{i,t}}$: The charging power of EV number i and time t
$\:{Q}_{INV}$: The inverter’s reactive power
$\:{P}_{INV}$: The inverter’s active power
$\:{S}_{INV}$: The inverter’s apparent power
RT: Real-time charging schedule
DT: Day-ahead charging schedule
$\:{P}_{Avg}^{T}$: The average load of the grid
$\:{P}_{t}^{Load}$: The base load of the grid at time t
$\:{P}_{t,m}^{EV}$: The charging power of EV number m at time t
$\:{f}_{1}^{{\prime\:}}$: Normalized value of f_1_
$\:{f}_{calc}$: Calculated value of f
$\:{f}_{max}$: Maximum value of f
$\:{f}_{min}$: Minimum value of f
$\:{f}_{1}^{{\prime\:}{\prime\:}}$: Normalized and scaled value of f_1_
